# *Mecp2* knock-out astrocytes affect synaptogenesis by interleukin 6 dependent mechanisms

**DOI:** 10.1016/j.isci.2024.109296

**Published:** 2024-02-23

**Authors:** Elena Albizzati, Martina Breccia, Elena Florio, Cecilia Cabasino, Francesca Maddalena Postogna, Riccardo Grassi, Enrica Boda, Cristina Battaglia, Clara De Palma, Concetta De Quattro, Davide Pozzi, Nicoletta Landsberger, Angelisa Frasca

**Affiliations:** 1 Department of Medical Biotechnology and Translational Medicine, University of Milan, via F.lli Cervi 93, 20054 Segrate, Milan, Italy; 2 Department of Biomedical Sciences, Humanitas University, via Rita Levi Montalcini 4, 20072 Pieve Emanuele, Milan, Italy; 3 IRCCS Humanitas Research Hospital, via Manzoni 56, 20089 Rozzano, Milan, Italy; 4 Department of Neuroscience Rita Levi-Montalcini, University of Turin, 10126 Turin, Italy; 5 Neuroscience Institute Cavalieri Ottolenghi, Regione Gonzole 10, 10043 Orbassano, Turin, Italy; 6 Department of Biotechnology, University of Verona, Cà Vignal 1, 37134 Verona, Italy; 7 Division of Neuroscience, IRCCS San Raffaele Scientific Institute, via Olgettina 58, 20132 Milan, Italy

**Keywords:** Cell biology, Immunology, Neuroscience, Omics, Transcriptomics

## Abstract

Synaptic abnormalities are a hallmark of several neurological diseases, and clarification of the underlying mechanisms represents a crucial step toward the development of therapeutic strategies. Rett syndrome (RTT) is a rare neurodevelopmental disorder, mainly affecting females, caused by mutations in the X-linked methyl-CpG-binding protein 2 (*MECP2*) gene, leading to a deep derangement of synaptic connectivity. Although initial studies supported the exclusive involvement of neurons, recent data have highlighted the pivotal contribution of astrocytes in RTT pathogenesis through non-cell autonomous mechanisms. Since astrocytes regulate synapse formation and functionality by releasing multiple molecules, we investigated the influence of soluble factors secreted by *Mecp2* knock-out (KO) astrocytes on synapses. We found that *Mecp2* deficiency in astrocytes negatively affects their ability to support synaptogenesis by releasing synaptotoxic molecules. Notably, neuronal inputs from a dysfunctional astrocyte-neuron crosstalk lead KO astrocytes to aberrantly express IL-6, and blocking IL-6 activity prevents synaptic alterations.

## Highlights

• High levels of IL-6 secreted by *Mecp2* KO astrocytes negatively affect synapses
• IL-6 upregulation results from an altered astrocyte-neuron crosstalk in Rett syndrome
• Astrocytes from *Mecp2* heterozygous mouse cortices express high levels of IL-6

## Graphical abstract

**Figure:**
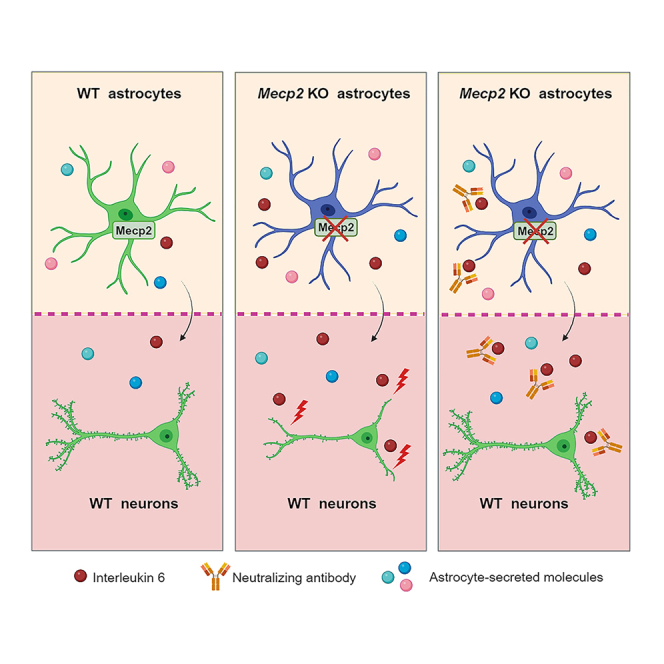

## Introduction

Astrocytes represent one of the most abundant classes of glial cells in the mammalian brain and play a pivotal role for proper health and function of the central nervous system (CNS), providing metabolic and trophic support to neurons.^1^ Accordingly, astrocyte-neuron interactions are fundamental for synaptic development across different brain regions since early embryonic stages to adulthood.^2^^,^^3^ By secreting bioactive proteins, such as neurotrophins, synaptogenic molecules, cytokines, and chemokines, astrocytes finely promote synaptic formation, functional maturation, and refinement.^3^^,^^4^^,^^5^^,^^6^ Consequently, altered secretion of these signals contributes to synaptic defects, as described for many neurodevelopmental, neuropsychiatric, and neurodegenerative disorders.^7^^,^^8^^,^^9^^,^^10^ Rett syndrome (RTT) is a rare neurodevelopmental disease caused in the vast majority of cases by mutations in the X-linked methyl-CpG-binding protein 2 (*MECP2*).^11^ Besides neurons, which suffer from severe morphological, functional, and synaptic defects,^12^^,^^13^^,^^14^^,^^15^ recent data indicated a role for astrocytes in RTT pathogenesis, reporting that *Mecp2* mutant astrocytes and their conditioned medium exert a negative effect on neuronal development, decreasing dendritic outgrowth and affecting overall maturation.^16^^,^^17^^,^^18^^,^^19^ The defective neuronal support by *MECP2* mutant astrocytes might be the result of several alterations, including molecular, morphological, metabolic, and functional defects.^19^^,^^20^^,^^21^ Accordingly, the sole loss of *Mecp2* in astrocytes is sufficient to cause pathological alterations, while rescuing *Mecp2* expression specifically in these glial cells greatly ameliorates RTT symptoms and leads to synaptic improvements, suggesting the possibility to target astrocytes for therapeutic purpose.^17^ Two proteomic studies analyzed deregulated proteins secreted by *Mecp2* knock-out (KO) astrocytes to disclose molecules influencing dendritic maturation. Ehinger and colleagues examined the secretome of KO cortical astrocytes isolated from P1-2 pups, revealing the decrease of Lcn2 and Lgals3, that, when added to null neurons, effectively improved dendritic arborization.^22^ More recently, Caldwell and collaborators analyzed the proteome of culture medium from KO cortical astrocytes isolated at P7 by immunopanning, reporting the alteration of several proteins, including an increase of Igfbp2 and BMP6, that were highlighted as molecular candidates involved in the occurrence of neuronal defects.^23^ However, the aforementioned studies analyzed the secretome of astrocytes cultured alone, without considering their crosstalk with neurons, which strongly affects molecular and functional properties of both cell populations,^24^^,^^25^^,^^26^ an issue particularly relevant for RTT, which is characterized by mosaic interactions between cells expressing either the wild type (WT) or mutant *MECP2* allele. Thus, in our study, we explored whether and how *Mecp2* KO astrocytes might alter synapses in WT neurons, investigating the astrocyte-neuron crosstalk in a co-culture system. We report that KO astrocytes secrete molecules that alter synaptogenesis in WT neurons and transcriptomic analyses suggested the involvement of inflammatory triggers. Accordingly, molecular investigations on KO astrocytes revealed an increase of interleukin-6 (IL-6) levels in the co-culture medium (CCM) as well as of its transcription, and we proved the causative role of this cytokine in synaptic impairments. Interestingly, excessive secretion of IL-6 exclusively occurs in the presence of WT neurons and lacks when KO astrocytes are cultured alone.

Overall, our study identifies a novel pathogenic mechanism triggered by the communication between WT neurons and *Mecp2* KO astrocytes that leads to synaptic alterations and might provide a novel therapeutic target for RTT and other *MECP2*-related disorders.

## Results

### Molecules secreted by *Mecp2* KO astrocytes affect synaptogenesis in WT neurons

**Figure 1:**
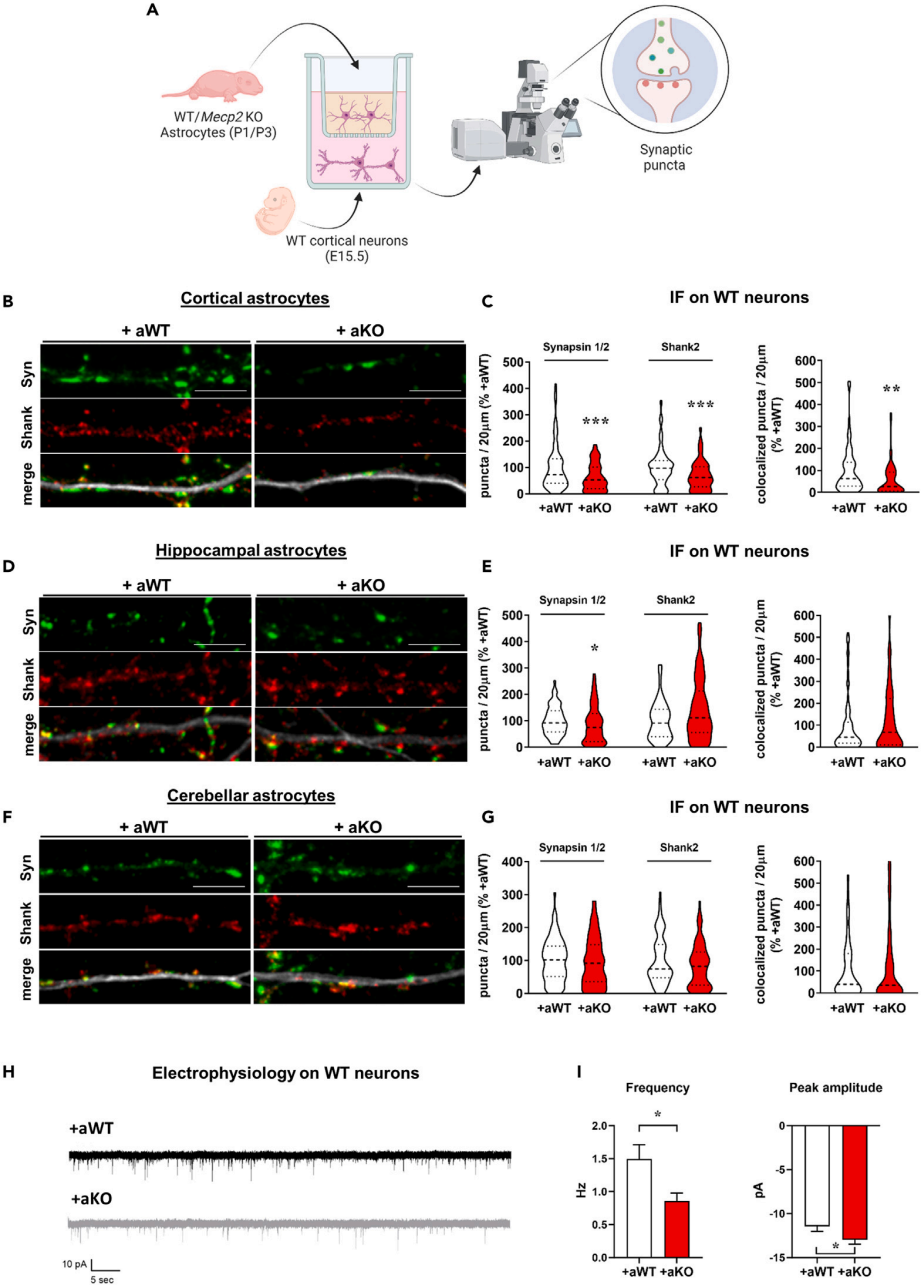
Soluble factors secreted by *Mecp2* KO astrocytes affect synaptogenesis, with cortical astrocytes showing the most detrimental effects (A) Experimental design overview. (B, D, and F) Representative images of primary branches from WT neurons (DIV14) immunostained for Synapsin1/2 (green), Shank2 (red) and their merge with MAP2 (white), in co-culture with cortical (B), hippocampal (D) and cerebellar astrocytes (F). Scale bar = 5 μm. (C, E, and G) Violin plots indicate the median (dashed line) and 25^th^ and 75^th^ percentiles (dotted lines) of Synapsin1/2, Shank2 and colocalized puncta density. Values for puncta number are expressed as percentages compared to WT-WT co-cultures (set at 100%). ∗p < 0.05, ∗∗p < 0.01, ∗∗∗p < 0.001 by Mann-Whitney test. Analyses were performed on n > 60 neurons (in C), on n > 66 neurons (in E) and on n > 46 neurons (in G) per experimental group from N > 7 (in C and E) or N > 5 (in G) biological replicates. All data derived from at least 2 independent experiments. (H) Representative traces of miniature excitatory post-synaptic currents (mEPSCs) recorded in neurons cultured with either WT or KO astrocytes. (I) Quantitative analysis of frequency and amplitude of mEPSCs. Data are represented as mean ± SEM. ∗p < 0.05 by Student’s t test. WT n = 27; KO n = 35.

Since astrocytes play key roles in synapse formation and RTT pathogenesis,^3^^,^^16^^,^^17^ we exploited a transwell-based co-culture between WT cortical neurons and *Mecp2* KO astrocytes to investigate the consequence of *Mecp2* deficiency in astrocytes on synaptogenesis. WT-WT co-cultures were used as control. The impact on synaptogenesis was assessed by analyzing the density of pre- and post-synaptic puncta, and their colocalization, as an index of functional synapses (Figure 1A). WT neurons matured under the influence of KO cortical astrocytes displayed reduced number of both pre- and post-synaptic puncta as well as of colocalized puncta, compared to control (Figures 1B and 1C), with no change in size (Figure S1). Of note, KO astrocytes cultured in close contact with WT neurons caused reduction of pre-synaptic puncta density, as well as of puncta area (Figure S2), thus suggesting that other factors beyond secreted molecules might alter the synaptic phenotype.

According to the heterogeneous properties of astrocytes depending on cerebral region, we assessed whether *Mecp2* deficiency diversely affects their synaptogenic potential when derived from brain regions other than the cortex.^27^ Interestingly, WT cortical neurons cultured with hippocampal KO astrocytes exhibited a selective impairment of the density of pre-synaptic puncta, with no effect on post-synapses and colocalization (Figures 1D and 1E), while cerebellar astrocytes did not cause any synaptic defect (Figures 1F and 1G).

In line with the morphological analysis, electrophysiological recording of excitatory synaptic basal transmission showed that neurons co-cultured with KO cortical astrocytes display a significant reduction of miniature excitatory post-synaptic currents’ (mEPSCs) frequency if compared to neurons with WT astrocytes. To note, a slight significant increase of mEPSC amplitude was observed in neurons with KO astrocytes, whereas the passive properties were not changed (Figures 1H, 1I, and S3).

These results demonstrate that molecules secreted by *Mecp2* KO cortical astrocytes impair synaptogenesis, therefore supporting a non-cell autonomous influence on WT neurons. The release of neurotoxic factors and/or defective secretion of synaptogenic molecules might be involved. Moreover, this effect is brain-area specific, as astrocytes from other brain areas, including the hippocampus and cerebellum, did not show the same capacity to influence synaptogenesis, pointing to cortical astrocytes as the most affected cells.

### Transcriptional profile of WT neurons confirms the detrimental action of *Mecp2* KO astrocytes on synapse formation, highlighting the role of pro-inflammatory cues

**Figure 2:**
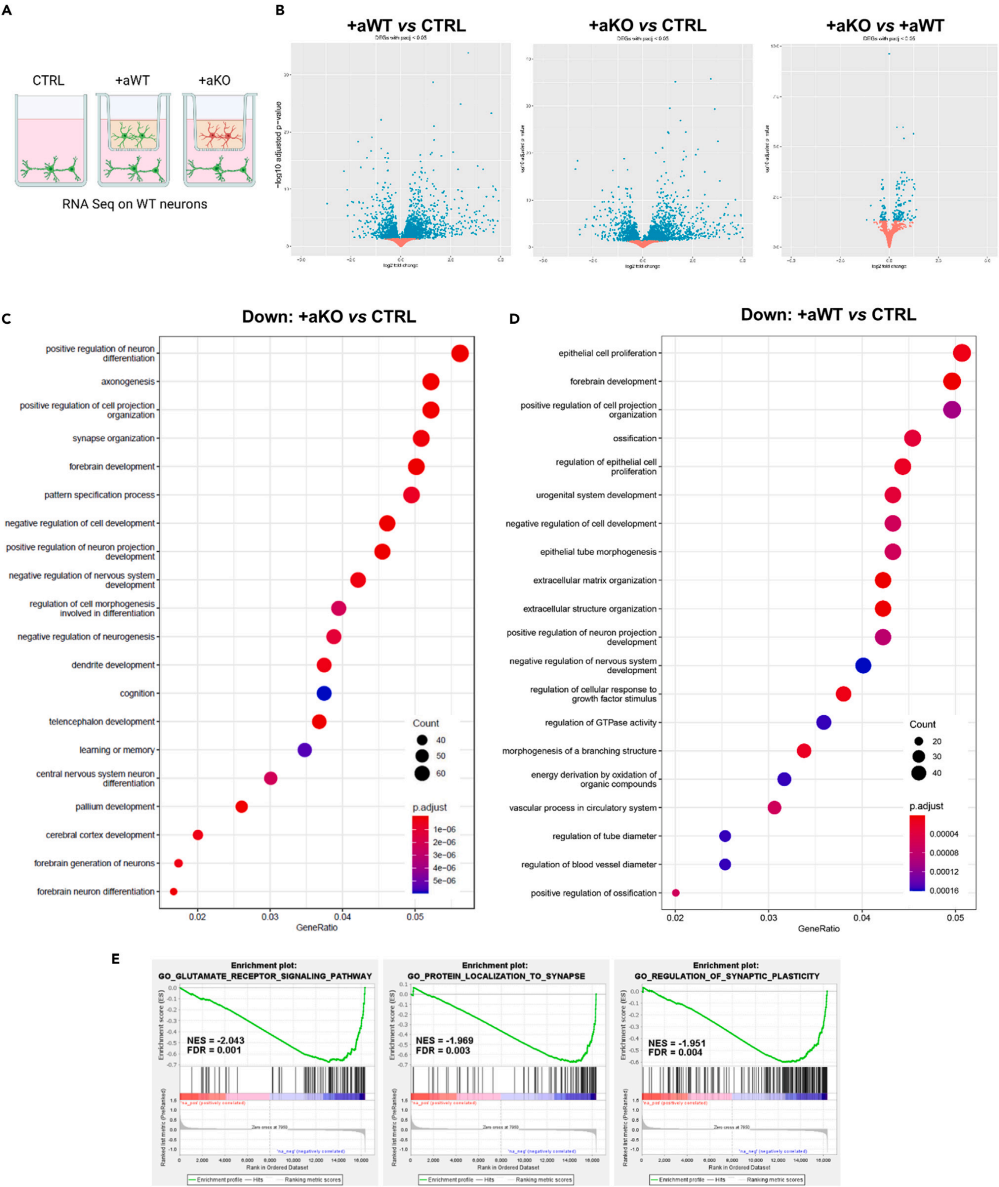
*Mecp2* KO astrocytes affect the expression of genes involved in synaptic maturation in WT co-cultured neurons (A) Experimental groups included in RNA-seq analysis: neurons cultured alone (CTRL) (n = 5) and neurons matured in co-culture with WT astrocytes (+aWT) (n = 7) or *Mecp2* KO astrocytes (+aKO) (n = 8). Replicate samples were derived from 2 independent experiments. (B) Volcano plots of DEGs with p.adj<0.05 from the different comparisons, plotted as log_2_FC against -log_10_ adjusted p value. Each dot represents a gene: top-right sector highlights genes that are significantly upregulated, top-left sector genes that are significantly downregulated, with p.adj < 0.05. (C and D) Enrichment analysis of downregulated DEGs at p.adj < 0.05 in +aKO vs*.* CTRL (C) and +aWT vs*.* CTRL (D) comparisons, showing the top 20 significant GO terms (biological processes). Size and color of dots represent number of genes associated with each term and FDR adjusted p value, respectively, according to the scale indicated in the figure. (E) Enrichment plots from preranked GSEA of the comparison +aKO vs*.* +aWT. Three of the top ten negatively correlated gene sets are reported, together with their normalized enrichment score (NES) and significance (FDR) (see Table S6 for complete results).

To gain insights into the effects mediated by cortical astrocytes on neurons, we performed bulk RNA-seq on WT neurons cultured with astrocytes, which were plated on transwell inserts (Figure 2). Transcriptomic analysis was conducted on neurons derived from three experimental groups, in which neuronal cells were cultured alone (“CTRL”), with WT astrocytes (“+aWT”), or with *Mecp2* KO astrocytes (“+aKO”) (Figure 2A). Transcriptional profiles of +aWT or +aKO neurons were compared to each other (+aKO versus +aWT) and to that of CTRL (+aWT versus CTRL; +aKO versus CTRL). Principal component analysis (PCA) of whole transcriptome expression highlighted that neurons from co-culture systems clustered together with respect to CTRL (Figure S4A), confirming that neurons dramatically change their transcriptional profiles when cultured with astrocytes.^24^ Indeed, both +aWT and +aKO showed a great number of differentially expressed genes (DEGs) when compared to CTRL, whereas few significant DEGs emerged from their comparison (Figures 2B and S4B; Tables S1–S3).

In order to identify the main biological processes influenced by the different conditions, gene ontology (GO) enrichment analysis was performed separately on downregulated or upregulated DEGs. Considering downregulated DEGs, pathways related to axonogenesis, synapse organization, dendritic development, and cognition emerged as the most affected in the comparison between +aKO versus CTRL (Figure 2C). Interestingly, the same pathways were not present in the comparison +aWT versus CTRL (Figure 2D), suggesting that KO astrocytes release synaptotoxic paracrine signals.

Notably, although a comparison between downregulated DEGs of +aKO versus +aWT was possible only using a padj < 0.1, processes related to synapse organization and activity appeared as the most affected ones (Figure S4C; Table S4). Collectively, these results highlighted the impact of *Mecp2* deficient astrocytes on proper synaptogenesis and neuronal maturation, thereby validating the immunofluorescence data.

To better investigate the overall molecular pathways affected in WT neurons by KO astrocytes, a preranked gene set enrichment analysis (GSEA) was conducted on the comparison +aKO versus +aWT. The analysis yielded several negatively correlated gene sets and only few positively correlated gene sets (NES<0 or >0, respectively, combined with FDR q < 0.05, Table S6). Among the top ten negatively correlated gene sets, we found “*glutamate receptor signaling pathway*,” “*protein localization to synapse*,” and “*regulation of synaptic plasticity*,” thus corroborating the morphological and functional alterations observed at synaptic level (Figure 2E).

**Figure 3:**
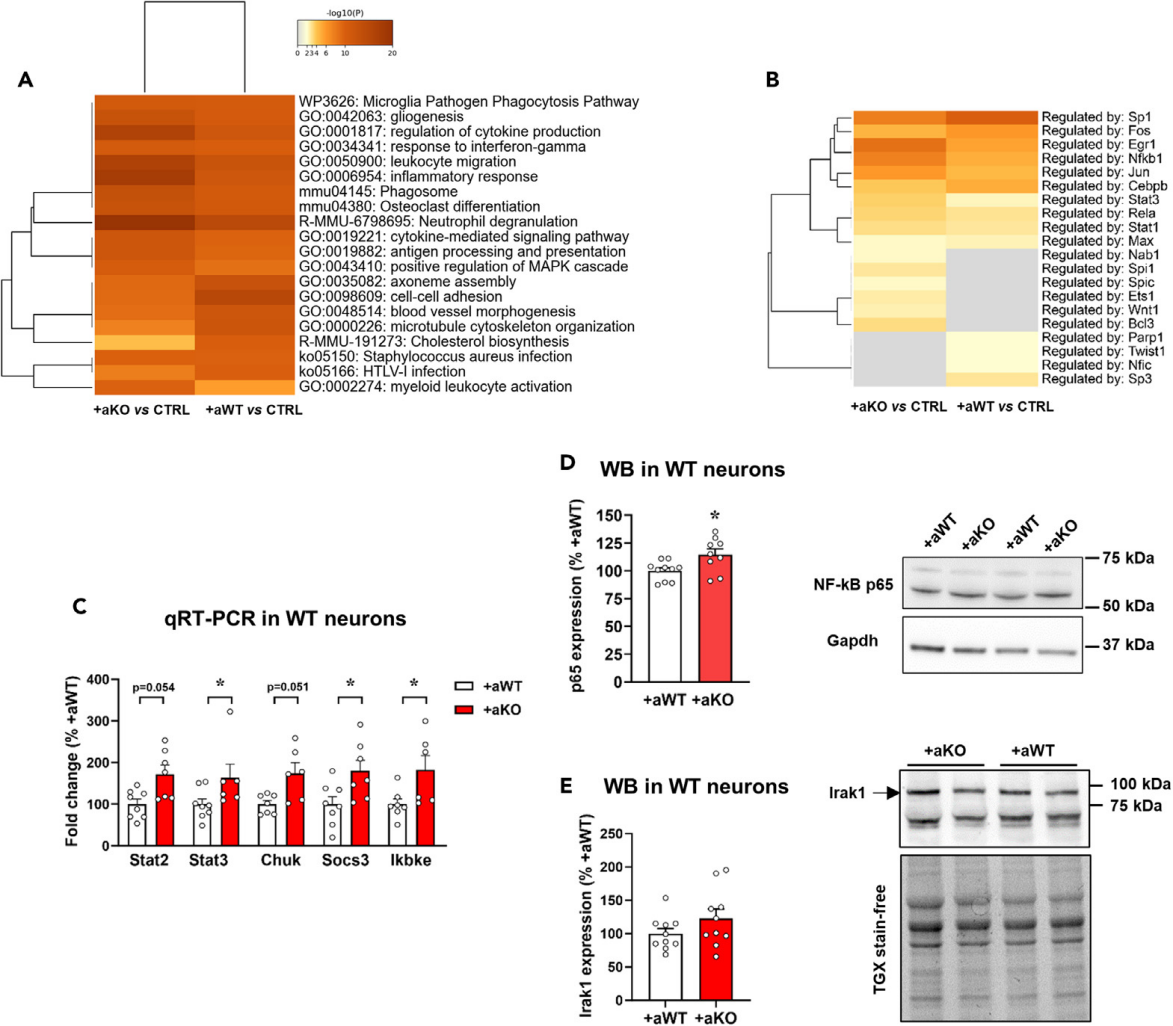
Inflammatory pathways are activated in WT neurons in culture with *Mecp2* KO astrocytes (A and B) Metascape analyses of upregulated pathways (see Tables S7 and S8) from +aWT vs*.* CTRL and +aKO vs*.* CTRL. Top 20 most represented biological processes together with their statistical value (represented as -log10 p value) (A) and enrichment analysis of transcriptional regulators using the TRRUST database (B) are shown. (C) The histogram reports the mRNA levels of a panel of genes associated with inflammatory responses. WT neurons (DIV14) cultured with KO astrocytes (+aKO, n = 6–7) were compared to WT neurons cultured with WT astrocytes (+aWT, n = 7–8). Data are represented as mean ± SEM and expressed as percentage of +aWT. ∗p < 0.05, by Mann-Whitney test. (D and E) Western blot analysis of NF-kB p65 protein levels (D) and Irak1 (E) in WT neurons (at DIV14) cultured with *Mecp2* KO or WT astrocytes (n = 10 +aWT and n = 9–10 +aKO). Data are represented as mean ± SEM and expressed as percentage of +aWT. Representative bands for p65 and Irak1 are reported above the corresponding graph. p65 and Irak1 signals were normalized using Gapdh and total protein content, respectively. Samples derived from 3 independent experiments.

Since the direct comparison of +aWT vs*.* +aKO did not provide any mechanistic suggestion for the onset of synaptic alterations, we focused on the comparison of +aWT vs*.* CTRL and +aKO vs*.* CTRL, by exploring the upregulated pathways derived from the GO analyses (Figure S4D; Tables S7 and S8). Interestingly, many biological processes associated with cytokines production/signaling and immune response were enriched in both GO analyses. To better extrapolate the entity of inflammatory pathways, we exploited Metascape resources on upregulated DEGs,^28^ finding that responses to cytokines and inflammatory stimuli were more represented in the +aKO vs*.* CTRL with respect to the +aWT vs*.* CTRL comparison (Figure 3A). Moreover, enrichment analysis of transcriptional regulators using the TRRUST database confirmed a stronger activation of transcriptional factors involved in inflammatory response (such as NFkB1, Jun, Stat3, Stat1, Spi1, Ets1, Wnt1, and Bcl3) in the +aKO vs*.* CTRL comparison (Figure 3B).

To confirm transcriptomic data, quantitative reverse transcription PCR (qRT-PCR) analysis was performed in WT neurons cultured with KO astrocytes, showing an upregulation of genes associated with inflammatory response, including Stat2, Stat3, Chuk, Socs3, and Ikbke (Figure 3C). Furthermore, western blot analysis indicated that protein expression of the inflammatory trigger p65 subunit of NF-kB was significantly increased in neurons cultured with KO astrocytes (Figure 3D). Conversely, the protein levels of Irak1, another component of the NF-kB pathway, that is consistently upregulated in *Mecp2* KO cerebral cortex,^29^ were not significantly modified (Figure 3E).

Overall, these data further confirm a defective role of *Mecp2* KO astrocytes in supporting synaptogenesis and point toward a possible role of inflammatory cues’ secretion as a candidate molecular mechanism.

### *Mecp2* KO astrocytes secrete excessive IL-6 and its synthesis depends on neuronal signals

**Figure 4:**
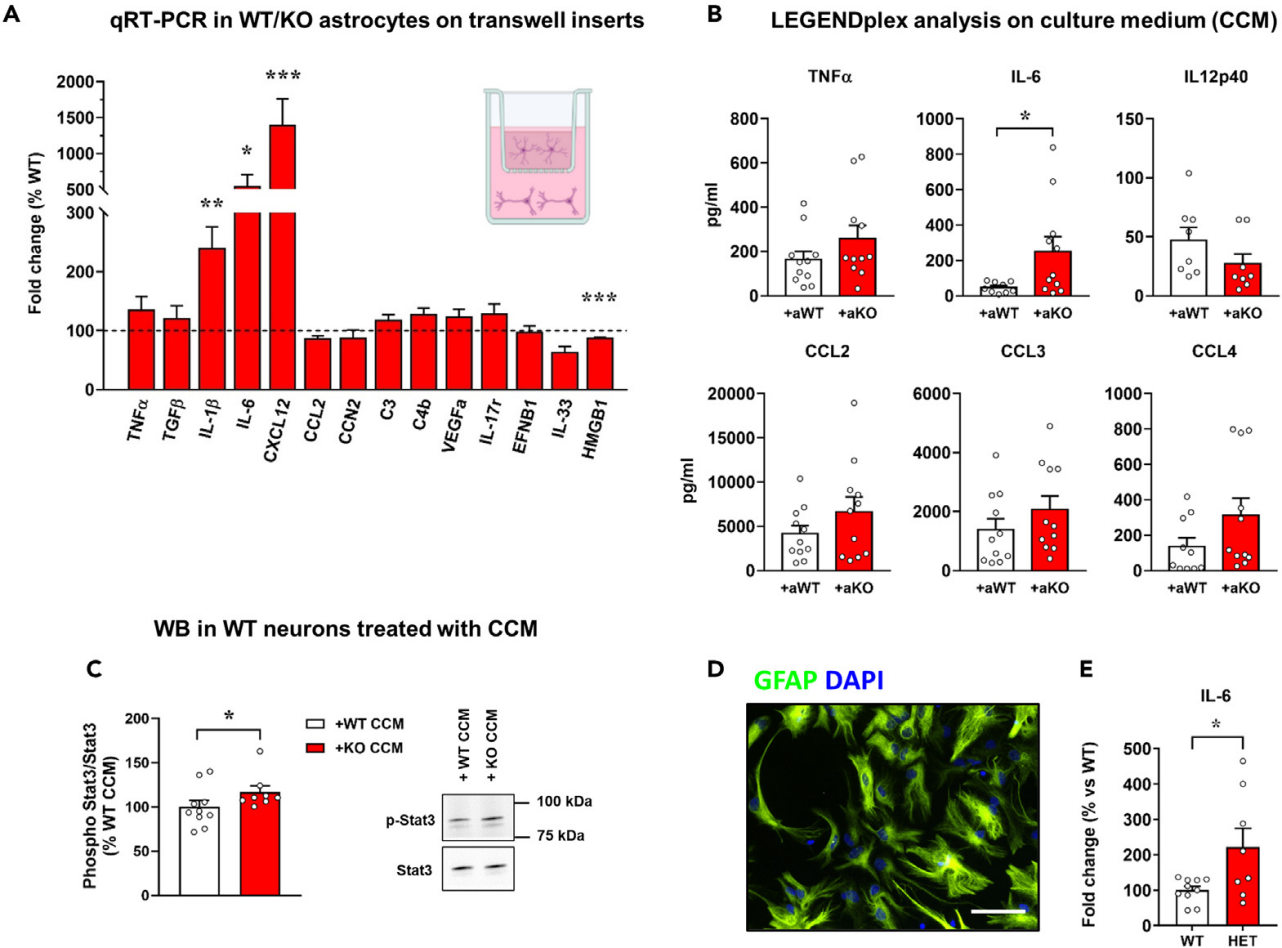
*Mecp2* KO astrocytes secrete excessive IL-6, when in culture with WT neurons (A) The graph depicts the mRNA expression of selected astrocyte genes in *Mecp2* KO cortical astrocytes cultured for 14 days with WT neurons. Data are expressed as percentages of +aWT condition (n = 10) and represented as mean ± SEM. ∗p < 0.05, ∗∗p < 0.01, ∗∗∗p < 0.001 by Student’s t test or Mann-Whitney test in accordance with data distribution. Samples derived from at least 3 independent experiments. (B) Cytokines’ quantification in the co-culture medium by LEGENDplex assay kit. Data are represented as mean ± SEM and expressed as percentage of +aWT condition (n = 11). ∗p < 0.05 by Student’s t test. Samples derived from at least 3 independent experiments. (C) Western blot analysis of phosphorylated Stat3 over Stat3 protein levels in WT neurons treated for 24 h with the medium derived from the co-cultures of neurons with WT (WT CCM) or KO astrocytes (KO CCM). Data are represented as mean ± SEM and expressed as percentage of +aWT (n = 9/10). Representative bands are reported. (D) Immunofluorescence staining for GFAP in acutely sorted astrocytes from P7 cortices, confirming the purity of astrocyte isolation. Scale bar = 30 μm. (E) The graph shows the mRNA expression of IL-6 in HET cortical astrocytes, compared to WT astrocytes. Data, expressed as percentage of WT samples, are reported as mean ± SEM. Samples (n = 10 WT and n = 9 HET) derived from 3 independent litters. ∗p < 0.05 by Student’s t test.

To confirm the activation of an inflammatory-like phenotype in KO astrocytes co-cultured with WT neurons, we measured mRNA levels of a panel of pro-inflammatory molecules in astrocytes cultured with neurons, along with their concentration in the CCM. qRT-PCR showed a strong upregulation of IL-1β, IL-6, and CXCL12 in KO astrocytes. Conversely, the expression of HMGB1, a trigger of inflammation in many neurodegenerative diseases, was slightly reduced (Figure 4A). In parallel, protein levels of the secreted cytokines and chemokines were measured by a cytometric bead-based immunoassay platform, finding a 3-fold increase in IL-6 concentration in the CCM derived from co-cultures with KO astrocytes, and a slight but not statistically significant increase of TNFα, CCL2, CCL3, and CCL4 (Figure 4B).

To verify that KO astrocytes are the main source of secreted IL-6, we measured its mRNA levels also in co-cultured WT neurons. Notably, IL-6 transcripts in neuronal cells were too low to be detected; however, by exploiting our RNA-seq data we did not reveal any deregulation of its expression in co-cultured neurons (Table S1). These results attribute excessive IL-6 secretion exclusively to KO astrocytes.

To assess that KO astrocytes secrete functional IL-6, we analyzed phosphorylation levels of the transcription factor Stat3 in neurons treated for 24 h (from DIV13 to DIV14) with the CCM derived from the co-cultures of WT astrocytes with WT neurons or KO astrocytes with WT neurons. Coherently with flow cytometry data, western blot analysis reported a significant increment of Stat3 phosphorylation in neurons treated with the KO CCM (Figure 4C).

Since the presence of serum in culture might predispose astrocytes toward a reactive phenotype, IL-6 levels were assessed in astrocytes directly isolated from a mouse model of RTT. To this aim, by taking advantage of MACS technology, astrocytes were acutely isolated from the cortex of P7 heterozygous (HET) animals and the corresponding WT female littermates at P7 (Figure 4D), and by qRT-PCR we observed a significant increase of IL-6 mRNA levels in HET astrocytes (Figure 4E). Interestingly, Pearson correlation analysis reported that IL-6 transcriptional levels tend to inversely correlate with the expression of wild-type *Mecp2* (r^2^ = 0.4341, p = 0.075), suggesting that its upregulation might be confined to Mecp2^*-*^ astrocytes.

**Figure 5:**
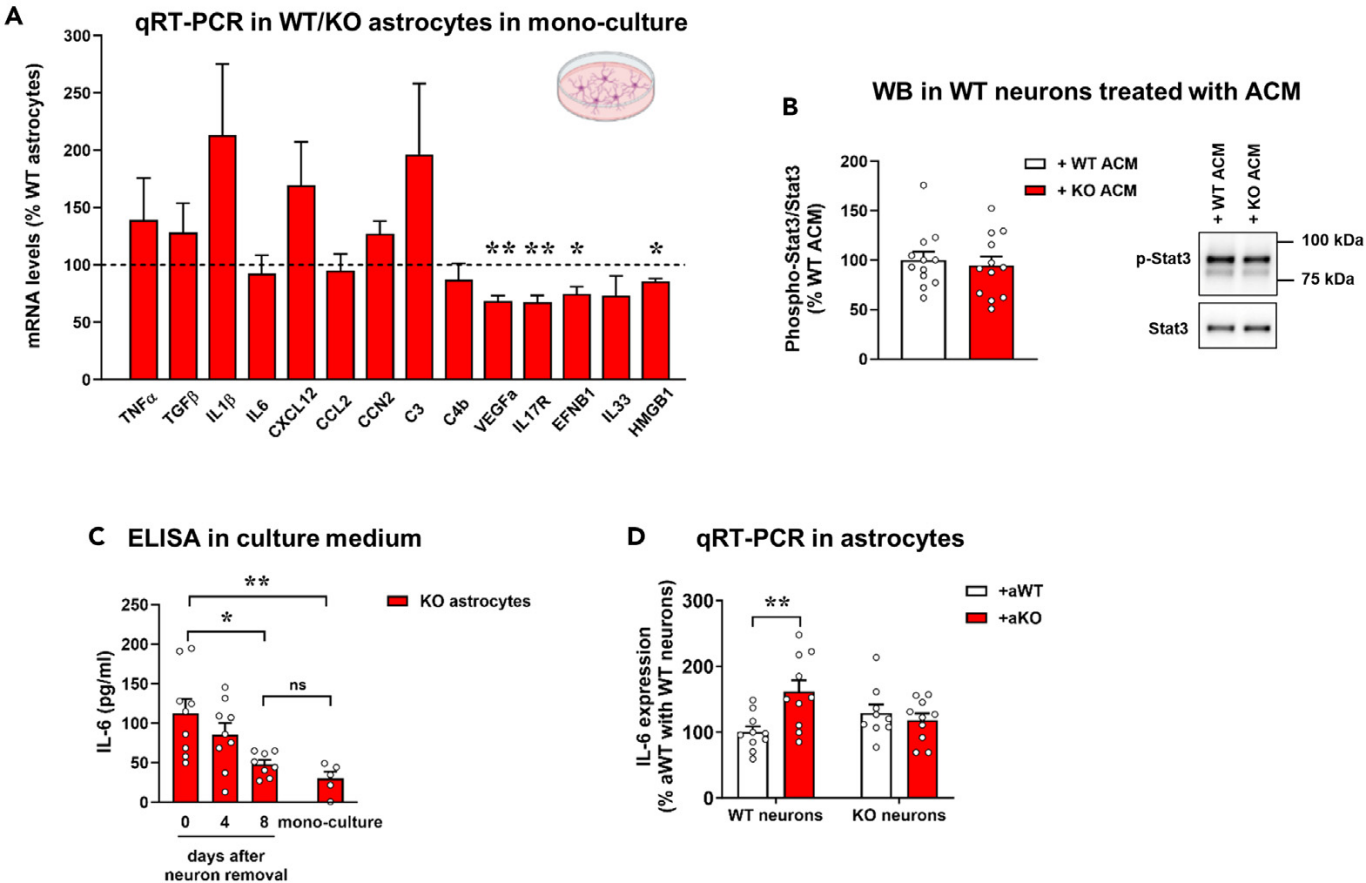
IL-6 secretion by *Mecp2* KO astrocytes depends on neuron-derived inputs (A) The graph depicts the mRNA levels of selected astrocyte genes in *Mecp2* KO cortical astrocytes cultured alone, expressed as percentages of WT astrocytes (n = 10). Data are represented as mean ± SEM. ∗p < 0.05, ∗∗p < 0.01 by Student’s t test or Mann-Whitney test in accordance with data distribution. (B) Western blot analysis of phosphorylated Stat3 over Stat3 protein levels in neurons exposed to WT or KO ACM. Data, represented as mean ± SEM, are expressed as percentages of neurons treated with WT ACM (n = 12); representative bands for phosphorylated and total Stat3 are reported. (C) The histograms report the IL-6 concentrations detected by ELISA assay in the medium of KO astrocytes at different days after removal of neurons (0, 4 and 8 days). Data are also compared to those analyzed in the medium of KO astrocytes in mono-culture. Data are represented as mean ± SEM. ∗p < 0.05, ∗∗ p < 0.01 by one-way ANOVA test, followed by Kruskal-Wallis test. (D) mRNA expression levels of IL-6 in WT and KO astrocytes when in culture with WT or KO neurons are reported as mean ± SEM, and expressed as percentage of WT-WT co-cultures (n = 9–11). ∗∗p < 0.01 by two-way ANOVA test, followed by Tukey’s post hoc test.

We next proceeded studying whether aberrant IL-6 secretion by KO astrocytes occurs in a cell-autonomous manner. For this purpose, we initially tested if KO astrocyte conditioned medium (ACM) negatively affects synapses. In line with previous publications,^16^^,^^18^^,^^19^ neurons treated with WT ACM showed a healthy phenotype in terms of pre- and post-synaptic puncta number, with a trend toward an increase compared with neurons treated with the control medium. On the contrary, KO ACM caused a detrimental effect on both synaptic compartments. In addition, the lack of any effect in neurons treated with heat-inactivated KO ACM (KO ACM∗) demonstrated that secreted neurotoxic factors are thermolabile (Figure S6). However, KO astrocytes cultured alone did not express higher levels of inflammatory molecules. Indeed, qRT-PCR revealed that the expression of IL-6, as well as that of IL-1β and CXCL12, was unaffected, whereas mRNA levels of VEGF, IL17R, EFNB1, IL33, and HMGB1 were downregulated in KO astrocytes (Figure 5A). Accordingly, the lack of Stat3 activation in neurons acutely treated with KO ACM excluded the involvement of IL-6 in the ACM-induced synaptic defects (Figure 5B).

To additionally explore whether IL-6 secretion by KO astrocytes is tightly dependent on the presence of neuronal inputs, we measured by ELISA its levels in the culture medium after neuron removal. In detail, astrocytes were maintained in culture with neurons for 14 days and then transwell inserts were transferred into new plates together with their original co-culture medium. IL-6 was quantified in the medium at DIV14 (before neuronal removal), and then 4 and 8 days after removal of neurons. As expected, we confirmed increased IL-6 secretion when neurons and astrocytes were still in co-culture (at DIV14), whereas a progressive decline of IL-6 concentration was observed after neuron removal. In particular, after 8 days, IL-6 levels were significantly decreased and returned to the values measured in the medium of KO astrocytes cultured alone (Figure 5C).

Furthermore, we assessed the influence of neuronal genotype on IL-6 expression, by analyzing its transcription in WT and KO astrocytes cultured with KO neurons. qRT-PCR data reported that *Mecp2* KO astrocytes aberrantly express IL-6 only when sensing WT neurons (Figure 5D), an evidence that strengthens the relevance of studying astrocyte-neuron communication in RTT, that is characterized by a mosaic combination of cells expressing either the WT or mutant *MECP2* allele.

### Aberrant IL-6 secretion by *Mecp2* KO astrocytes causes dendritic and synaptic alterations

**Figure 6:**
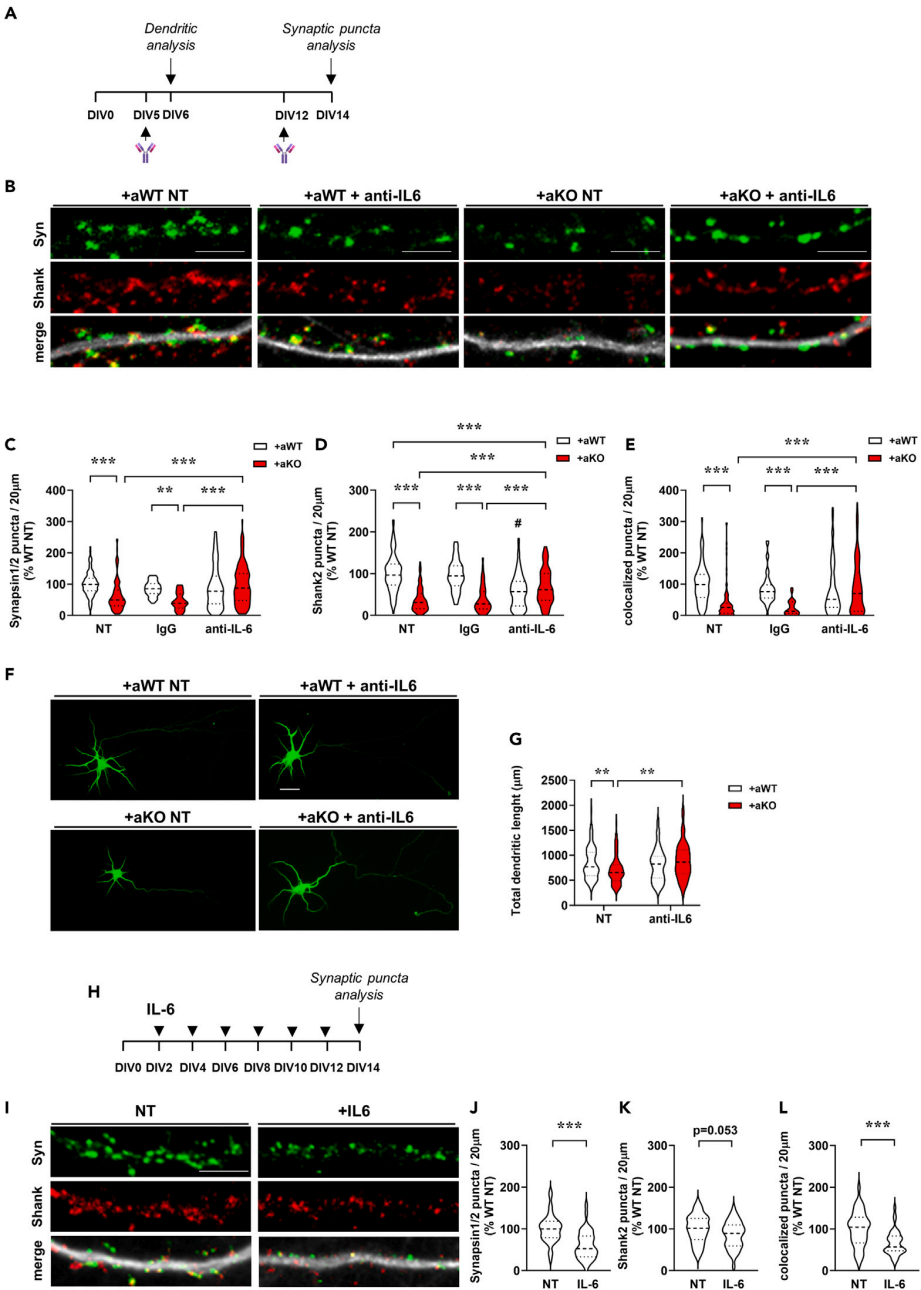
IL-6 released by *Mecp2* KO astrocytes causes dendritic and synaptic defects in WT neurons (A) Neutralizing anti-IL6 antibody, or an isotypic antibody, was added to co-cultures and analysis of dendrites and synaptic puncta density was conducted at DIV6 and DIV14, respectively. (B) Representative images of primary branches from WT neurons (DIV14) immunostained for Synapsin1/2 (green), Shank2 (red) and their merge with MAP2 (white). Scale bar = 5 μm. (C‒E) Violin plots indicate the median (dashed line) and 25^th^ and 75^th^ percentiles (dotted lines) of Synapsin1/2 (C), Shank2 (D) and colocalized puncta number (E) in neurons co-cultured with WT (+aWT) or KO (+aKO) astrocytes seeded on transwell inserts. Values for puncta number are expressed as percentages of +aWT untreated condition (NT). ∗∗p < 0.01, ∗∗∗p < 0.001 by two-way ANOVA test followed by Tukey’s post hoc test. #p < 0.001 denotes the statistical comparison between +aWT condition treated with anti-IL-6 antibody versus untreated (NT) or IgG-treated +aWT neurons. Analyses were performed on n > 50 neurons per experimental group from N > 4 biological replicates. (F) Representative images of Map2 positive neurons (DIV6), when cultured with WT or KO astrocytes and treated with anti-IL6 antibody or left untreated (NT). Scale bar, 20 μm. (G) Violin plot depicts the total dendritic length in WT neurons cultured with WT (+aWT) or KO (+aKO) astrocytes, following treatment with anti-IL6 antibody or left untreated (NT). Analysis was performed on n > 40 neurons per experimental group from N = 5 replicates. ∗∗p < 0.01 by two-way ANOVA test, followed by Tukey’s post hoc test. (H) Recombinant IL-6 (200 pg/mL) was added to WT cortical neurons every 2 days, starting at DIV2. The arrowheads indicate the time of IL-6 treatment. (I) Representative images of primary branches from WT neurons (DIV14) immunostained for Synapsin1/2 (green), Shank2 (red) and their merge with MAP2 (white). Scale bar, 5 μm. (J‒L) Violin plots indicate the median (dashed line) and 25^th^ and 75^th^ percentiles (dotted lines) of Synapsin1/2 puncta density (J), Shank2 puncta density (K) and puncta colocalization (L) of WT neurons treated with recombinant IL-6. Data are indicated as mean ± SEM. Analyses were performed on n = 40 NT and n = 41 treated neurons from N = 4 biological replicates. ∗∗∗p < 0.001 by Student's *t* test.

To evaluate whether the excessive secretion of IL-6 contributes to the observed synaptic defects, an anti-IL6 antibody was added to co-cultures (Figure 6A). We found that inhibiting the IL-6 signaling rescued the number of pre-synaptic terminals, whereas post-synaptic puncta were only partially recovered. Importantly, the same treatment significantly increased the number of colocalized puncta, therefore restoring functional synapses. The specific effect of the blocking antibody against IL-6 was verified by testing an isotypic IgG antibody, which did not produce the same beneficial effects. In line with the physiological role of IL-6 in synaptic formation and maintenance,^30^ blocking IL-6 in neurons cultured with WT astrocytes reduced the density of post-synaptic puncta (Figures 6B–6E; DIV14). Of interest, cytokine blockade also rescued the well-known dendritic alterations induced by KO astrocytes in cultured neurons^16^^,^^18^ (Figures 6F and 6G; DIV6).

Complementary, to evaluate the direct action of IL-6 on neurons, the recombinant cytokine at the dosage deduced by flow cytometry (200 pg/mL) was added to pure WT neuronal cultures every two days, starting from DIV2 (Figure 6H). Immunofluorescence analyses of synaptic puncta indicated that IL-6 led to a significant reduction of the density of pre-synaptic puncta and colocalized puncta, and an almost significant defect in the post-synaptic puncta (Figures 6I‒6L).

## Discussion

In this study, we investigated astrocyte-neuron communication in the context of RTT by analyzing the ability of *Mecp2* KO astrocytes to regulate synapse formation via paracrine signals. In agreement with literature, our results confirmed the damaging action exerted by *Mecp2* KO astrocytes on neuronal health, corroborating the importance of *Mecp2* expression in astrocytes to sustain neuronal maturation by non-cell autonomous mechanisms.^16^^,^^18^^,^^31^ In addition, by analyzing the density of synaptic puncta, which is profoundly compromised in RTT,^13^^,^^14^^,^^15^ we demonstrated that *Mecp2* KO cortical astrocytes negatively affect synaptogenesis. Furthermore, we disclosed that molecules secreted by KO astrocytes lead to an abnormal neuronal inflammatory response, triggered by IL-6. Indeed, IL-6 blockade by a neutralizing antibody restores dendritic and synaptic alterations, thus indicating the detrimental action of this cytokine, whose aberrant expression in astrocytes requires a crosstalk with neurons.

Several groups have highlighted the heterogeneity of astrocyte populations and how astrocytes from different brain areas exert distinct effects on neuronal functions.^32^ Our results suggest that cortical KO astrocytes may contribute to the altered synaptic density observed in RTT and this evidence is in line with our recent data demonstrating that cortical glial cells show the most severe cytoskeletal and transcriptional alterations in *Mecp*2 KO brain.^33^

In the last years, the release of toxic molecules by KO astrocytes has been proposed as a potential mechanism responsible for neuronal defects in RTT and many attempts have been conducted to disentangle such a mechanism with the final goal to develop *ad hoc* therapeutic strategies. Diversely from previous studies examining the molecular phenotypes of KO astrocytes alone,^21^^,^^22^^,^^23^ in order to identify the molecules involved in the occurrence of synaptic defects, we deployed an alternative strategy aimed at assessing deregulated pathways in neurons cultured with astrocytes to deduce upstream regulators. The validity of this approach is supported by the significant downregulation of pathways associated with synaptic maturation and functionality, in line with our immunofluorescence results. Further, the observation that synapse-related pathways were downregulated in neurons cultured with KO astrocytes compared to neurons alone suggested that KO astrocytes release synaptotoxic molecules, although a defective secretion of synaptogenic cues cannot be excluded. Indeed, we believe that a synergic cooperation between reduced secretion of synaptogenic factors and increased release of toxic molecules (among which we identified IL-6) might contribute to the observed synaptic defects.

The novelty of our study also relies on the evidence that the neuronal-astrocyte crosstalk impinges on the *Mecp2* KO astrocyte secretome. Indeed, our molecular data indicated a significant upregulation of IL-6 in KO astrocytes only when maintained in communication with WT neurons, while a decline of IL-6 secretion was observed when neurons were removed. In agreement, previous transcriptomic and proteomic studies performed in KO astrocytes cultured alone never reported an increase of IL-6,^21^^,^^22^^,^^23^ indicating the importance of astrocyte-neuron communication for dictating the molecular and functional properties of astrocytes. Although mainly neglected in the RTT field, a very recent paper considered the crosstalk between human astrocytes and neurons. The authors reported that the morphological alterations of RTT astrocytes stem from a combination of intrinsic and non-cell autonomous defects, whereas the alterations in gene expression are caused by astrocyte autonomous mechanisms.^19^ In contrast, we found that the capacity of neurons to modulate IL-6 expression in *Mecp2* KO astrocytes depends on the genotype of neuronal cells. The observation that increased expression of IL-6 selectively occurs in KO astrocytes when cultured with WT neurons and not with KO ones reinforces the importance of neuronal inputs for the regulation of astrocyte properties.^24^ Molecular mechanisms underpinning this fine regulation might rely on neuronal activity and many neuronal-derived signals, including neurotransmitters and neuromodulators,^26^ an aspect that deserves further investigations. Intriguingly, these data suggest that molecular differences exist between full KO and heterozygous mosaic tissue, recalling the “cell interference hypothesis” proposed for Pcdh19 mutation, by which the co-existence of Pcdh19-positive and negative cells might affect neuronal interactions.^34^

The upregulation of IL-6 in astrocytes isolated from the cortex of P7 heterozygous animals further strengthens its involvement in RTT and excludes the possibility of methodological artifacts of culturing cells in a medium supplemented with serum. In addition, although in the present work we have not discriminated Mecp2^*+*^ from Mecp2^*-*^ astrocytes, the trend of an inverse correlation between *IL-6* and *Mecp2* expressions suggests an increased production of the cytokine by astrocytes expressing the mutant allele.

IL-6 is a cytokine involved in the regulation of neuronal and synaptic functions,^30^ and, as occurs for this family of proteins, it exhibits pleiotropic actions depending on its concentration, displaying either neurotrophic properties or detrimental activity.^35^ Indeed, several data are available regarding the effects of IL-6 on excitatory synapse formation,^30^ which might depend on the source of IL-6 and its concentration. For instance, a recent paper has pointed out that a transient IL-6 elevation at early step of neuronal development promotes glutamatergic synaptogenesis.^36^ On the other hand, a chronic production of IL-6 by astrocytes at mature stages led to detrimental neuronal effects, including severe synapse loss and degeneration.^37^ In our experimental conditions, we proved the synaptotoxic effects of IL-6 by reporting that the neutralizing antibody against IL-6 can significantly improve synaptic alterations. This antibody binds IL-6 with picomolar affinity, thus inhibiting its biological functioning. Complementary, a chronic treatment with IL-6 on WT neurons led to an impaired synaptic phenotype, confirming the synaptotoxic action of IL-6 on developing cortical neurons. Our results support the importance of a fine regulation of IL-6 levels as demonstrated by the evidence that an abnormal upregulation of its release by KO astrocytes alters the process of synaptic formation in neurons. At the same time, even a decreased IL-6 level in WT co-cultures affects the same process suggesting a protective role of this cytokine.

The relevance of our findings is emphasized by previously published data. First, numerous clinical and experimental data point to the presence of a subclinical inflammatory status in RTT, characterized by cytokine dysregulation and aberrant NF-kB pathway.^29^^,^^38^^,^^39^^,^^40^^,^^41^ Of note, IL-6 increased expression has already been described in the brain, saliva, and plasma of patients suffering from RTT,^39^^,^^42^ as well as in other neuropsychiatric disorders.^43^ Notably, IL-6 has been associated with blood brain barrier (BBB) dysfunction^44^ and, recently, a defective BBB integrity has been reported in RTT animal models.^45^ Additionally, IL-6 overexpression in the mouse brain correlates with neurological abnormalities^37^^,^^46^ and, conversely, its inhibition improves social behaviors.^47^ The relevance of IL-6 and its pathway for mental diseases is highlighted by the observation that they have been already proposed for the development of novel therapies, which however should aim at attenuating IL-6 activity without completely abolishing it, considering the aforementioned physiological roles.^47^^,^^48^

In conclusion, our study identified IL-6 as a synaptotoxic molecule triggered by *Mecp2* KO astrocytes, providing an interesting therapeutic target for RTT and other *MECP2*-related disorders.

### Limitations of the study

This study is focused on the molecular characterization of the crosstalk between *Mecp2* KO astrocytes and neurons mediated by secreted factors. However, physical interactions between cells, and not only secreted molecules, concur to cell-to-cell communication, particularly during development. Indeed, besides producing many synaptic modulators, astrocytes also express several adhesion proteins, which mediate astrocyte-synapse interactions at tripartite synapses. Thus, future studies are required, in the RTT field, to investigate the molecular mechanisms activated in cultures where astrocytes and neurons mature in contact, with the final aim to detect other molecules, beyond IL-6. Considering the heterogeneity properties of astrocytes, we have to mention that the heterotypic co-cultures that we used allowed to compare the synaptogenic potential of region-specific astrocytes.^27^ However, we cannot exclude that this setting could have masked other alterations proper of the respective homotypic culture. Importantly, an investigation of IL-6 secretion by human-derived RTT astrocytes might reinforce the clinical relevance of our results.

## STAR★Methods

### Key resources table

**Table:** 

REAGENT or RESOURCE	SOURCE	IDENTIFIER
**Antibodies**		

Chicken polyclonal anti-Synapsin1/2 IgY fraction	Synaptic Systems	Cat# 106 006; RRID: AB_2622240
Mouse monoclonal anti-Shank2 purified IgG	Synaptic Systems	Cat# 162 211; RRID: AB_2661874
Rabbit monoclonal anti-MAP2 (D5G1) XP® antibody	Cell Signaling	Cat# 8707; RRID: AB_2722660
Mouse monoclonal anti-GFAP (clone GA5)	Merck Millipore	Cat# MAB3402; RRID: AB_94844
Goat anti-Chicken IgY (H+L) Cross-Adsorbed Secondary Antibody, Alexa Fluor™ Plus 488	ThermoFisher Scientific	Cat# A32931; RRID: AB_2762843
Donkey anti-Mouse IgG (H+L) Highly Cross-Adsorbed Secondary Antibody, Alexa Fluor™ 488	ThermoFisher Scientific	Cat# A21202; RRID: AB_141607
Donkey anti-Mouse IgG (H+L) Highly Cross-Adsorbed Secondary Antibody, Alexa Fluor™ 568	ThermoFisher Scientific	Cat# A10037; RRID: AB_2534013
Donkey anti-Rabbit IgG (H+L) Highly Cross-Adsorbed Secondary Antibody, Alexa Fluor™ 647	ThermoFisher Scientific	Cat# A31573; RRID: AB_2536183
Mouse monoclonal anti-Irak1 antibody (F-4)	Santa Cruz Biotechnology	Cat# 5288; RRID: AB_2249106
Mouse monoclonal anti-NF-kB p65 (F-6) antibody	Santa Cruz Biotechnology	Cat# sc-8008; RRID: AB_628017
Mouse monoclonal anti-GAPDH antibody	Invitrogen	Cat# MA1-16757; RRID: AB_568547
Mouse monoclonal anti-STAT3 (124H6) antibody	Cell Signaling	Cat# 9139; RRID: AB_331757
Rabbit monoclonal anti-Phospho-Stat3 (Tyr705) (D3A7) XP® antibody	Cell Signaling	Cat# 9145; RRID: AB_2491009
HRP conjugated Goat polyclonal anti-Mouse IgG (H+L) antibody	Jackson ImmunoResearch	Cat# 115-035-003; RRID: AB_10015289
HRP conjugated Goat polyclonal anti-Rabbit IgG (H+L) antibody	Jackson ImmunoResearch	Cat# 111-035-144; RRID: AB_2307391
Rat monoclonal IgG1 anti-IL6 antibody, Clone # MP5-20F3	R&D Systems	Cat# MAB406; RRID: AB_2233899
Rat IgG1, kappa Isotype Control, Unconjugated, Clone R3-34 antibody	BD Biosciences	Cat# 554682; RRID: AB_395507

**Bacterial and virus strains**		

No bacterial and virus strains used	N/A	N/A

**Biological samples**		

No biological samples used	N/A	N/A

**Chemicals, peptides, and recombinant proteins**		

Recombinant Murine Interleukin-6	Peprotech	Cat# 216-16
Neurobasal™ Medium	Gibco™	Cat# 21103049
B-27™ Supplement (50x), serum free	Gibco™	Cat# 17504044
L-Glutamine solution	ThermoFisher Scientific	Cat# G7513
Penicillin/Streptomycin (P/S)	ThermoFisher Scientific	Cat# P0781
Dulbecco’s Modified Eagle’s Medium (DMEM), high glucose, pyruvate	Gibco™	Cat# 41966029
Ham’s F-10 Nutrient mix	Gibco™	Cat# 31550023
Fetal Bovine Serum (FBS), qualified, heat inactivated, E.U.-approved, South America Origin	ThermoFisher Scientific	Cat# 10500064
Hanks' Balanced Salt Solution (HBSS), no calcium, no magnesium, no phenol red	ThermoFisher Scientific	Cat# 14175095
HEPES solution 1 M, pH 7.0–7.6, sterile-filtered	Sigma-Aldrich	Cat# H0887
Sodium bicarbonate (NaHCO3)	Sigma-Aldrich	Cat# S6014
Trypsin-EDTA (0.25%) phenol red	ThermoFisher Scientific	Cat# 25200-056
Trypsin-EDTA (0.05%)	ThermoFisher Scientific	Cat# 25300054
Poly-D-lysine hydrobromide	Sigma-Aldrich	Cat# P7886
Dulbecco’s Phosphate Buffered Saline (DPBS), no calcium, no magnesium	Euroclone	Cat# ECB4004L
PureZOL RNA isolation Reagent	Bio-Rad	Cat# 7326890
Glycogen RNA grade	ThermoFisher Scientific	Cat# R0551
DNase I Amplification Grade	Sigma-Aldrich	Cat# AMPD1
15KU Deoxyribonuclease I type IV from bovine	Sigma-Aldrich	Cat# D5025
SYBR Selected Master MIX	Applied Biosystems	Cat# 4472908
Bovine Serum Albumin (BSA)	Sigma-Aldrich	Cat# A3059
Triton X-100	Sigma-Aldrich	Cat# T8787
DAPI	ThermoFisher Scientific	Cat# 62248
Fluoromount Aqueous Mounting Medium	Sigma-Aldrich	Cat# F4680
4x Laemmli Sample Buffer	Bio-Rad	Cat# #1610747
2-Mercaptoethanol	Sigma-Aldrich	Cat# M6250
Protease Inhibitor Cocktail (PIC)	Sigma-Aldrich	Cat# P8340
PhosSTOP Phosphatase Inhibitor Cocktail Tablets	Roche	Cat# 4906845001
Precision Plus Protein All Blue	Bio-Rad	Cat# 1610373
Tween® 20	Sigma-Aldrich	Cat# P1379
WESTAR SUN ECL	CYANAGEN	Cat# XLS063
WESTAR ANTARES ECL	CYANAGEN	Cat# XLS0142
WESTAR SUPERNOVA ECL	CYANAGEN	Cat# XLS3
Xtra Taq Pol RTL GL	GENESPIN	Cat# XSTS-T5XRTL GL
Xtra RTL GL Reaction Buffer 5X	GENESPIN	Cat# XSTS-T5XRTL GL
Deoxynucleotide Set, 100 mM	Sigma-Aldrich	Cat# DNTP100A

**Critical commercial assays**		

Phire animal tissue direct PCR kit	ThermoFisher Scientific	Cat# F140WH
Agilent RNA 6000 Nano Kit	Agilent Technologies	Cat# 5067-1511
TruSeq® Stranded mRNA Library Prep	Illumina	Cat# 20020594
KAPA Library Quantification Kit	KapaBiosystems	Cat# KK4824
RT^2^ First Strand Kit	QIAGEN	Cat# 330404
SsoAdvanced™ PreAmp Supermix	Bio-Rad	Cat# 1725160
LEGENDplex™ Mouse Cytokine Release Syndrome Panel	BioLegend	Cat# 741023
LEGENDplex™ Mouse Cytokine Panel 2	BioLegend	Cat# 740134
ELISA kit	Immunological Sciences	Cat# IK4209
Anti-GLAST(ACSA-1) MicroBead Kit human, mouse, rat	Miltenyi Biotec	Cat# 130-095-826

**Deposited data**		

Array Express Repository	This paper	ArrayExpress: E-MTAB-12393 https://www.ebi.ac.uk/biostudies/arrayexpress/studies/E-MTAB-12393?key=65ac7976-7b9c-4547-a5b8-845b4316548f

**Experimental models: Cell lines**		

No cell lines used	N/A	N/A

**Experimental models: Organisms/strains**		

*Mecp2tm1.1* Bird CD1 mouse strain	Laboratory of Nicoletta Landsberger at University of Milan	Cobolli Gigli et al.^49^

**Oligonucleotides**		

List of primers for genotyping: See Table S9	Metabion	N/A
List of primers for qRT-PCR: See Table S10	Metabion	N/A

**Recombinant DNA**		

No recombinant DNA used	N/A	N/A

**Software and algorithms**		

NIS-Elements	Nikon	https://www.microscope.healthcare.nikon.com/products/software/nis-elements RRID:SCR_014329
Leica Application Suite	Leica Microsystems	RRID:SCR_016555
Fiji/ImageJ	Fiji	https://imagej.nih.gov/ij/RRID:SCR_002285
GraphPad Prism 8	GraphPad Software LLC	https://www.graphpad.com RRID:SCR_002798
RStudio	RStudio	RRID:SCR_000432
Gene Set Enrichment Analysis	UC San Diego and Broad Institute	https://www.gsea-msigdb.org/gsea/index.jsp RRID:SCR_003199
Metascape	Metascape	https://metascape.org/gp/index.html#/main/step1 RRID:SCR_016620
Uvitec Nine Alliance Software	Uvitec Cambridge	https://www.uvitec.co.uk/
QuantStudio 5 Data Analysis Software	ThermoFisher Scientific	https://www.thermofisher.com/us/en/home/global/forms/life-science/quantstudio-3-5-software.html
BioRender	BioRender	https://biorender.com

**Other**		

Falcon™ Cell Strainers, 40 μm, Nylon	Corning	Cat# 352340
PDL coated coverslips	Neuvitro	Cat# GG-12-PDL
Thincert™ Cell Culture Inserts 6 Well plates, tc, transparent membrane (PET), pore diameter 0.4 μm	Greiner Bio-One	Cat# GR657641
Thincert™ Cell Culture Inserts 24 Well plates, tc, transparent membrane (PET), pore diameter 0.4 μm	Greiner Bio-One	Cat# GR662641
4-15% Criterion™ TGX Stain-Free™ Protein gel, 26 well, 15μL	Bio-Rad	Cat# 5678085
Trans-Blot® Turbo™ Midi Nitrocellulose Transfer Packs	Bio-Rad	Cat# 1704159
MACS Separation MS Columns	Miltenyi Biotec	Cat# 130-042201

### Resource availability

#### Lead contact

Further information and requests for resources should be directed to and will be fulfilled by the lead contact Frasca Angelisa, angelisa.frasca@unimi.it.

#### Materials availability

This study did not generate new unique reagents.

#### Data and code availability

• RNA-seq data in this study have been deposited in the ArrayExpress repository. They are publicly available as of the date of publication. https://www.ebi.ac.uk/biostudies/arrayexpress/studies/E-MTAB-12393?key=65ac7976-7b9c-4547-a5b8-845b4316548f.
• This paper does not report original code.
• Any additional information required about the data reported in this paper is available from the lead contact upon request.

### Experimental model and study participant details

#### Animals

The *Mecp2*^*tm1.1*^ Bird mouse strain was originally purchased from the Jackson Laboratories and then backcrossed and maintained on a clean CD1 background.^49^ These mice recapitulate the typical phenotype of C57BL/6 mice, with the advantage of having a larger progeny and minor risk of litter cannibalization. Mouse genotype was determined by PCR on genomic DNA purified from ear punch biopsies, and both KO male and HET female animals were used for different experiments. Mice were housed in a temperature- and humidity-controlled environment in a 12-h light/12-h dark cycle with food and water *ad libitum*. All procedures were performed in accordance with the European Union Communities Council Directive (2010/63/EU) and Italian laws (D.L.26/2014). Protocols were approved by the Italian Council on Animal Care in accordance with the Italian law (Italian Government decree No. 210/2017 and 187/2022).

#### Primary cultures of neurons

Primary cortical neurons were obtained from WT and *Mecp2* null mouse embryos. When only WT neurons are prepared, embryos were generated by mating WT females with WT male mice. Conversely, *Mecp2* HET females were mated with WT male mice to generate both WT and KO embryos. The day of vaginal plug was considered E0.5 and primary neurons were prepared from E15.5 embryos to avoid glial contamination. Embryos were sacrificed by decapitation, brains were removed under a microscope and rapidly immersed in ice-cold HBSS. Meninges were gently removed, and cerebral cortex from both hemispheres was rapidly dissected into small pieces and maintained in cold HBSS until tissue dissociation. Tissues were washed in HBSS, incubated with 0.25% trypsin/EDTA for 7 min at 37°C and the digestion was blocked with dissociation medium (DMEM HG containing 10% FBS and 1% Pen/Strep). Then, cortices were accurately washed and mechanically dissociated by gently pipetting. Cells were counted (Countess Automated Cell Counter, ThermoFisher) and, depending on experimental needs, neurons were plated on poly-D-lysine-coated plates (0.1 mg/mL), poly-D-lysine-coated glass coverslips (1 mg/mL) or directly on astrocytes, at the density described below.

#### Primary cultures of astrocytes

Primary cultures of astrocytes were prepared from cerebral tissue of P1-P3 WT and *Mecp2* KO mice (cerebellar astrocytes specifically from P3), generated by mating *Mecp2* HET females with WT male mice. Pups were decapitated and brains immediately collected on ice-cold HBSS. Meninges were carefully removed and, depending on the experiment, cortices, hippocampi or cerebella were isolated from both hemispheres and immersed in HBSS containing 10 mM HEPES, 4 mM Na_2_HCO_3_ and 1% P/S until dissociation. Mouse genotype was determined by PCR on genomic DNA purified with Phire animal tissue direct PCR kit. Tissues were then incubated in 0.25% trypsin/EDTA for 30 min at 37°C, mechanically dissociated in astrocyte culture medium (DMEM HG containing pyruvate plus 1:1 Ham’s F-10, 10% FBS and 1% Pen/Strep) with a glass Pasteur and filtered through cell strainers of 40 μm pore size to obtain a single cell suspension. The resulting cells were centrifuged at 1,500 g for 7 min, re-suspended in culture medium, and plated on poly-D-lysine-coated 75 cm^2^ flasks (15 μg/mL). At DIV4, flasks were shaked in astrocyte culture medium containing 10 mM HEPES at 200 rpm for 8 h at 37°C to eliminate other cell types, and the medium was replaced with fresh culture medium. Cells were incubated in a humidified incubator at 37°C and 5% CO_2_ and culture medium was refreshed every 3–4 days. When astrocytes reached confluence (DIV12-DIV15), they were washed in D-PBS to remove death cells or cell debris, and then detached by 0.25% trypsin/EDTA diluted in HBSS (1:2 ratio) and counted in a Bürker chamber. Depending on experimental needs, they were plated on poly-D-lysine-coated plates (15 μg/mL), poly-D-lysine-coated glass coverslips (1 mg/mL) or transwell membrane inserts, at the density described below.

#### Isolation of mouse cortical astrocytes

To perform gene expression analysis on astrocytes acutely sorted from P7 *Mecp2* HET mice and the corresponding WT female littermates, animals were sacrificed by rapid decapitation and brains collected in ice-cold HBSS. Meninges were carefully removed and cortices from both hemispheres were isolated and immersed in HBSS containing 10 mM HEPES, 4 mM NaHCO_3_ and 1% P/S until dissociation. Tissues were then incubated in 0.05% trypsin/EDTA containing DNase I (1:1,000) for 5 min at 37°C, mechanically dissociated by gently pipetting and filtered through cell strainers of 40 μm pore size adding two volumes of astrocyte culture medium (DMEM plus 1:1 Ham’s F-10, 10% FBS and 1% P/S). Cell suspensions were then centrifuged at 1,000 rpm for 10 min at 4°C. Positive selection of astrocytes was performed by magnetic labeling of ACSA1-positive cells, by using a biotin-conjugate anti-ACSA1 antibody and the following labeling with streptavidin-coated magnetic beads. Labeled cells were retained by ferromagnetic columns placed in a magnetic field.^50^ Finally, cells were directly lysed in 1 mL PureZOL and stored at −80°C until RNA extraction.

### Method details

#### Astrocyte-neuron co-cultures

##### In contact co-cultures

Astrocytes (30,000 cells) were plated on glass coverslips and after 2–4 days, neurons were added on the top of astrocyte monolayers, at low density (10,000 cells/well). Cells were cultured, during the first 48 h, in a 5% FBS co-culture medium (Neurobasal medium containing 5% FBS, 2% B27, 1% L-Glutamine, 1% P/S), then in a medium containing lower serum concentration (Neurobasal medium containing 2.5% FBS, 2% B27, 1% L-Glutamine, 1% P/S) for the entire duration of the experiment, corresponding to 14 days for neurons. Co-cultures were filled with fresh medium at DIV7 for a third of the original volume.

##### Transwell co-cultures

Astrocytes were plated at a density of 8,000 cells/transwell membrane inserts for 24-well plates, 60,000 cells/transwell membrane inserts for 12-well plates or of 150,000 cells/transwell membrane for 6-well plates. Neurons (derived from 3 different embryos per experiment) were plated at a density of 15,000 or 30,000 cells/glass coverslip for 24-well plates, 60,000 cells/glass coverslip for 12-well plates or 200,000 cells/well for 6-well plates. Inserts with astrocytes were maintained in their culture medium for 4 days, with a change of the medium after 2 days to remove cells that did not attach. Then, medium was replaced with neuron culture medium and inserts were carefully transferred above neurons ∼1 h after seeding. The transwell-based co-culture was maintained until DIV7 or DIV14. At DIV7, both neurons and astrocytes were filled with fresh neuron culture medium for a third of the original volume, except when treated with IL-6 neutralizing or isotypic antibody (see the specific section). At DIV14, co-culture medium (CCM) was collected and stored at −80°C.

#### Astrocyte conditioned medium (ACM) preparation

For ACM preparation, astrocytes were plated at a density of 100,000 cells/well in 6-well plates. When confluent, they underwent starvation by removing FBS from medium. Briefly, after washing cells twice in DMEM (a rapid wash, followed by an incubation of 90 min), the medium was replaced by neuronal culture medium without B27 (Neurobasal containing 1% L-Glutamine and 1% P/S). After 48 h, ACM was collected and centrifuged at 1,200 rpm for 5 min at 4°C to remove cell debris. A cocktail of proteases inhibitor was added 1:1,000 to each sample and ACM was stored at −80°C until use.

#### Neuronal treatment

##### Treatment with the neutralizing antibody

Transwell based co-cultures were treated with either neutralizing antibody for IL-6 (0.5 mg/mL) or an isotypic antibody Rat IgG_1_ (1 mg/mL), diluted 1:500 and 1:1,000, respectively, in the final volume of culture medium (Neurobasal containing 2% B27, 1% L-Glutamine, 1% P/S). Treatment was performed at DIV5 for dendritic length analysis and at DIV5 and DIV12 for synaptic puncta analyses. Untreated co-cultures were filled with an equal volume of culture medium. In addition, at DIV5, transwell inserts were filled with either 50 μL (when in 24-well plates) or 100 μL (when in 6-well plates) of medium.

##### Treatment with recombinant IL-6

WT cortical neurons (30,000 cells/coverslip) were treated with recombinant IL-6 (200 pg/mL) every 2 days, starting from DIV2. Neurons were analyzed at DIV14.

##### Treatment with ACM or CCM

Neurons (30,000 cells/coverslip) were incubated with pre-warmed (37°C) ACM or co-culture medium (CCM) at DIV13 for 24 h. Heat inactivation of ACM was performed by boiling it at 95°C for 5 min and slowly cooling it down to 37°C prior to neuronal treatment. Neuronal culture medium was added as control on neurons (NT).

#### Electrophysiology

Patch-clamp recordings on primary cell cultures were performed as previously reported.^36^ In brief, recordings were carried out in Krebs-Ringer HEPES (KRH) as extracellular solution with the following composition (in mM): 125 NaCl, 5 KCl, 1.2 KH_2_PO_4_, 1.2 MgSO_4_, 2 CaCl_2_, 25 HEPES, and 6 Glucose; pH 7.4. The basal glutamatergic synaptic transmission recorded as miniature excitatory potential synaptic currents (mEPSCs) were detected in the presence of Bicuculline (20 μM) and Tetrodotoxin (TTX; 1 mM) using the following internal solution (in mM): 139 K-gluconate, 10 KCl, 2 MgCl_2_, 10 HEPES-Na^+^, 1 EGTA, 4 ATP, 0.2 GTP; pH 7.2. Glass pipettes of 4–6 MΩ were used as recording electrodes, pulled with a vertical puller (Narishige PC-10) by a 2-steps temperature protocol (step 1: 51,9°C; step 2: 44,1°C).

The recordings were performed in whole-cell configuration at −70 mV as holding potential. The electrical signals were amplified by a Multiclamp200B (Axon instruments), filtered at 2 kHz, digitized at 20 kHz with a DIGIDATA 1440 and stored with pClamp 10 (Axon instruments). Access resistance was continuously monitored during the experiment and cells with an access resistance >20 MΩ were discarded from the analysis.

#### RNA sequencing and bioinformatic analyses

Primary neurons were lysed in PureZOL and RNA was extracted following manufactures instructions. Samples were incubated with DNase at 37°C for 15 min to remove eventual genomic DNA contaminations. Then, enzymatic reaction was inactivated in PureZOL and a second RNA extraction was performed.

Generation of RNA-Seq data was performed by GENARTIS srl (Verona, Italy), as follows. RNA purity was measured at a NanoDrop Spectrophotometer (ThermoFisher Scientific) and RNA integrity was assessed using the RNA 6000 Nano Kit on a Bioanalyzer (Agilent Technologies). All samples showed an RNA integrity number (RIN) > 9. RNA samples were quantified using the Qubit RNA BR Assay Kit (ThermoFisher Scientific). RNA-Seq libraries were generated using the TruSeq stranded mRNA kit (Illumina) from 400 ng of RNA samples, after poly(A) capture and according to manufacturer’s instructions. Quality and size of RNA-Seq libraries were assessed by capillary electrophoretic analysis with the Agilent 4200 Tape station (Agilent Technologies). Libraries were quantified by real-time PCR against a standard curve with the KAPA Library Quantification Kit (KapaBiosystems, Wilmington, MA, USA). Libraries were pooled at equimolar concentration and sequenced on a NovaSeq6000 (Illumina) generating >20 million fragments in 150PE mode for each sample. Sequencing read trimming and quality of reads were assessed using FastQC software (http://www.bioinformatics.babraham.ac.uk/projects/fastqc/). Starting from raw FASTQ files, the first 10 nt of read2 were trimmed as they presented a lower quality than expected (Q < 30). In addition, reads with more than 10% of undetermined bases or more than 50 bases with a quality score <7 were discarded. Reads were then clipped from adapter sequences using Scythe software (v0.991) (https://github.com/vsbuffalo/scythe), and low-quality ends (Q score <20 on a 10-nt window) were trimmed with Sickle (v1.33) (https://github.com/vsbuffalo/sickle). Filtered reads were aligned to the Mouse reference genome GRCm38 (Ensembl release 102) using STAR (v2.7.6a) with default parameters and quantMode TranscriptomeSAM option that output alignments translated into transcript coordinates. After reads mapping, the distribution of reads across known gene features, such as exons (CDS, 5′UTR, 3′UTR), introns and intergenic regions, was verified using the script read_distribution.py provided by RSeQC package (v3.0.1). Read counts on genes were quantified using RSEM (v.1.3.3) and Mouse Ensembl release 102 annotation. Genes-level abundance, estimated counts and gene length obtained with RSEM were summarized into a matrix using the R package tximport (v1.18.0) and, subsequently, the differential expression analysis was performed with DESeq2 (v1.30.0) integrating the “day of sample preparation” as variable in the model. To generate more accurate Log2 FoldChange estimates, the shrinkage of the Log2 FoldChange was performed applying the apeglm method.

Gene Ontology (GO) enrichment analysis was performed using clusterProfiler, an R Package for comparing biological themes among gene clusters^51^ (Bioconductor version: Release (3.18.0)). The function simplify was used to remove redundancy of enriched GO terms. Differentially expressed genes (DEGs) with p.adj<0.05 were included in the analysis. Only for the comparison of WT neurons cultured with WT or KO astrocytes, GO analysis was conducted with DEGs with p.adj<0.1. FDR adjusted p value (q-value) <0.05 was used as a threshold and GO terms fulfilling this condition were defined as significantly enriched.

Preranked Gene Set Enrichment Analysis (GSEA)^52^ (version 4.1.0, the Broad Institute of MIT and Harvard; https://www.gsea-msigdb.org/gsea/downloads.jsp) was performed on a pre-ordered gene list ranked according to the log2 fold changes from DESeq2, between WT neurons cultured with *Mecp2* KO astrocytes and WT neurons cultured with WT astrocytes. GSEA calculated a Normalized Enrichment Score (NES) of gene sets present in the MsigDB 7.2 (Molecular Signatures Database; https://www.gsea-msigdb.org/gsea/msigdb), with 1,000 permutations set to generate a null distribution for enrichment score. ‘c5.go.bp.v7.2.symbols.gmt’ was the gene set database used for enrichment analysis and FDR q-value<0.05 was defined as the cut-off criteria for significance. Metascape analyses were performed using the Metascape platform^28^ (version 3.5; https://metascape.org) and giving in input gene lists of upregulated DEGs with p.adj<0.05 of comparisons ‘+aWT’ versus CTRL (2493 DEGs, Table S2) and ‘+aKO’ versus CTRL (2890 DEGs, Table S3).

#### Quantitative reverse transcription PCR (qRT-PCR)

Total RNA was extracted from neurons cultured in 6-well plates, from astrocytes cultured on transwell membrane inserts or on 6-well plates, and from astrocytes sorted from P7 mouse cortices using PureZOL. RNA was quantified using a NanoDrop 1000 spectrophotometer (ThermoFisher Scientific) and its integrity verified by agarose gel electrophoresis. cDNA was synthesized using the RT^2^ First Strand Kit according to the manufacturer’s instructions and used as template for qRT-PCR with SYBR Green Master Mix and a QuantStudio 5 Real-Time PCR System (ThermoFisher Scientific). Due to the low amount of RNA extracted from astrocytes on transwell, a preamplification step was performed using SsoAdvanced PreAmp Supermix prior to qPCR, following manufacturer’s instructions. Melting curve showed a single product peak, indicating good product specificity. The best housekeeping gene was selected for each comparison among *Cyclophilin A*, *Rpl13*, *Hprt* and *Ywhaz*, and fold change in gene expression was calculated using the 2ˆ(-Delta Ct) method.

#### Immunofluorescence

Neurons (at DIV7 or DIV14) plated on glass coverslips were fixed in 4% paraformaldehyde (PFA) plus 10% sucrose for 8 min at RT and, after three washes in 10mM PBS, they were stored in 10 mM PBS containing 0.1% sodium azide at 4°C until staining. To assess the efficiency of MACS sorting, 10 μL of cell suspension was transferred on glass coverslips and cells stained for astrocytic marker. First, cells were washed in 10 mM PBS and then permeabilized in PBS containing 0.2% Triton X-100 for 3 min on ice. After three washes in PBS containing 0.2% BSA, blocking solution (4% BSA in PBS) was added for 15 min before incubating the primary antibodies (Synapsin1/2 1:500, Shank2 1:300, MAP2 1:1000 on neurons; GFAP 1:500 for astrocytes), diluted in PBS containing 0.2% BSA overnight (4°C). Then, cells were washed in PBS containing 0.2% BSA before incubation (1 h at RT) with Alexa Fluor secondary antibodies, diluted 1:500 in PBS containing 0.2% BSA. Several washes in 0.2% BSA in PBS were then performed and nuclei were stained with 1:1,000 DAPI in PBS solution for 10 min at RT. Finally, cells were washed in 10mM PBS before mounting the glass coverslips on glass microscope slides with Fluoromount Aqueous Mounting Medium.

#### Microscopy and image analysis

To analyze the total dendritic length, images of WT neurons (at DIV6) were acquired at Nikon Eclipse Ti at 20X. Dendritic length was evaluated using NeuronJ, a plugin of ImageJ. We measured the length of each dendrite for each neuron and the sum of such values defined the total dendritic length. To analyze synaptic markers, z stack images of synaptic puncta (1,024 × 1,024 pixel resolution) were acquired either at 1× digital zoom using an SR Apo TIRF 100× oil-immersion objective mounted on a Nikon Ti2 microscope equipped with an A1+ laser-scanning confocal system (16-bit grayscale depth images) or at 1.4× digital zoom using a 63× oil-immersion objective mounted on a Leica DMI3000B microscope equipped with an Sp5 laser-scanning confocal system (8-bit grayscale depth images), depending on the experiment. Acquisition of synaptic puncta was performed using a step size of 0.3 μm. For each dataset acquisition, parameters (offset background, digital gain, and laser intensity) were maintained constant among different experimental groups. By Fiji software, maximum intensity projection images were converted to binary images and processed with a fixed threshold for each channel acquired. Puncta density was calculated by counting only puncta lying along manually selected ROIs within 20 μm of 3 primary branches/neuron. Only puncta with a minimum size of 0.16 μm^2^ were counted using *Analyze Particles*.^53^ To assess puncta co-localization of pre- and post-synaptic markers, the Fiji Plugin *Colocalization highlighter* was run on each z stack image acquired. Co-localized puncta were quantified in manually selected ROIs of the binary mask created from the maximum intensity projection. Only puncta with a minimum size of 0.1 μm^2^ were counted. Sample size of neurons analyzed per experimental group was assessed based on previous work.^54^

#### Western blot

To perform Western blots on neurons, cells were rapidly washed with DPBS at RT and then lysed by a cell scraper in sample buffer containing 2-Mercaptoethanol (1:10 v/v), protease (1:200 v/v) and phosphatase (1:10 v/v) inhibitors and sonicated at 30 Hz for 5 s. After denaturation at 70°C for 5 min, equal volume of each sample (15 μL) was resolved on 4–15% Criterion TGX Precast Protein Gels. Before transfer, a Stain-Free gel image was acquired using a UV-transilluminator (ESSENTIAL V6 System, UVITEC Ltd, UK) and used for relative quantification. Proteins were blotted on a nitrocellulose membrane using the *Trans*-blot SD (Bio-Rad) semidry apparatus and ponceau S staining was used to verify proper protein transfer. Membranes were then incubated at RT for 1 h in blocking solution (Tris-buffered saline containing 0.5% Tween 20 (TBST) and 5% non-fat milk or 5% BSA) before adding primary antibodies at the proper dilution: anti-NF-kB p65 (1:500 in 5% milk-TBST); anti-IRAK1 (1:1000 in 5% BSA-TBST); anti-phospho-Stat3 (1:1,000 in 5% BSA-TBST); anti-Stat3 (1:2,500 in 5% BSA-TBST); anti-Gapdh (1:10,000 in 5% milk-TBST). After 3 washes in TBST, blots were incubated with the appropriate HRP-conjugated secondary antibody diluted 1:10,000 in 5% milk or 5% BSA-TBST. Immunocomplexes were visualized using the ECL substrates kits from Cyanagen and imaged on ALLIANCE MINI HD9 system (UVITEC Ltd, UK). Quantification of bands was performed using the Uvitec Nine Alliance Software, with the relative density of bands corrected on background signal. P65 and Irak1 signals were normalized on Gapdh and total protein, by using TGX-stain free gel, respectively. Phospho-Stat3 levels were normalized to total Stat3 levels. Percentages plotted on graphs were calculated by normalizing all conditions to the control group.

#### Cytokines quantification

Cytokines were detected in culture media by FRACTAL Unit (Flow cytometry Resource, Advanced Cytometry Technical Applications Laboratory, San Raffaele Scientific Institute, Milan) with two LEGENDplex assay kits: LEGENDplex Mouse Cytokine Release Syndrome Panel and LEGENDplex Mouse Cytokine Panel 2 according to the manufacturer's instructions. Cell culture media were used undiluted. Samples were acquired using a BD FACSCantoTM II Cell Analyzer (BD Biosciences) using BD FACS Diva software and equipped with three lasers: blue (488 nm), red (633 nm) and violet (405). FCS files were analyzed by using LEGENDplex Data Analysis Software (BioLegend) according to the manufacturer’s instructions, obtaining the absolute concentration for each molecule.

#### ELISA assay for IL-6

KO astrocytes, plated on transwell inserts, were maintained in cultures with WT neurons for 14 days (corresponding to neurons DIV14). At this time point, astrocytes were transferred into new plates with the original medium but in the absence of neurons, and maintained in cultures for other 8 days. Immediately after neuronal removal and 4 and 8 days later, an aliquot of medium was collected from each well and frozen at −80°C, in the presence of protease inhibitor (1:1000). By ELISA assay, IL-6 concentration was quantified according to the manufacturer’s instruction. IL-6 concentration was also calculated in KO ACM.

### Quantification and statistical analysis

Data are expressed as mean ± SEM, except for violin plots in which the bar indicates the median with interquartile range. Replicates are indicated in figure legends. Before any statistical analysis, normality distribution was evaluated for each dataset by D’Agostino and Pearson test and outliers were assessed by ROUT test (Q = 1%) or Grubb’s test (a = 0.05%). Unpaired Student’s t-tests or Mann–Whitney tests were used for the comparisons of two groups, in accordance with data distribution. To assess the effect of ACM treatment on WT neurons, Kruskal–Wallis test followed by Dunn’s post hoc test was applied. Statistical significance for multiple group comparisons was determined by two-way ANOVA, followed by Tukey’s post hoc test. All statistical analyses were performed using Prism 8 (GraphPad Software, La Jolla, CA, United States). A p value <0.05 was considered significant. ∗p < 0.05, ∗∗p < 0.01, ∗∗∗p < 0.001.
