# Supplementary material for: Mecp2 knock-out astrocytes affect synaptogenesis by interleukin 6 dependent mechanisms
Source: iScience. 2024 Feb 23;27(3):109296. doi: 10.1016/j.isci.2024.109296 (PMC10926209; doi:10.1016/j.isci.2024.109296)
Supplement: Document S1. Figures S1–S6 [file mmc1.pdf]

iScience, Volume *27*

Supplemental information

Mecp2 knock-out astrocytes affect synaptogenesis

by interleukin 6 dependent mechanisms

Elena Albizzati, Martina Breccia, Elena Florio, Cecilia Cabasino, Francesca Maddalena Postogna, Riccardo Grassi, Enrica Boda, Cristina Battaglia, Clara De Palma, Concetta De Quattro, Davide Pozzi, Nicoletta Landsberger, and Angelisa Frasca

Figure S1

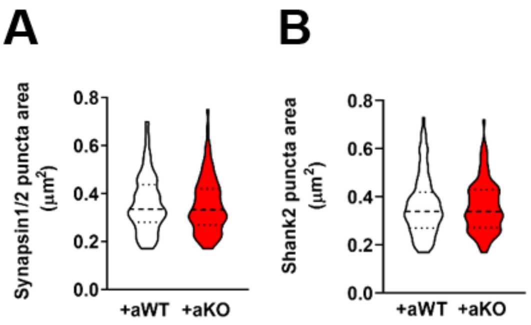

Figure S1. Soluble factors secreted by *Mecp2* KO astrocytes do not affect puncta size, related to Figure 1.
Violin plot indicates the median (dashed line) and 25th and 75th percentiles (dotted lines) of Synapsin1/2 (A) and Shank2 (B) puncta area of neurons co-cultured with WT or KO cortical astrocytes seeded on transwell inserts. Data are represented as mean±SEM. Analyses were performed on n>107 neurons from N>15 biological replicates. Samples derived from 4 independent experiments.

Figure S2

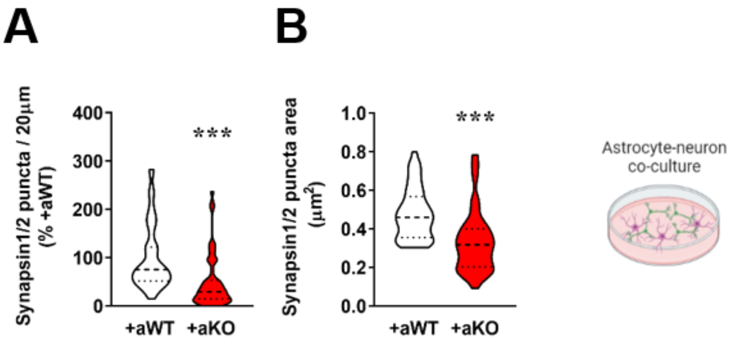

Figure S2. *Mecp2* KO astrocytes induce a significant reduction of pre-synaptic puncta number and size when cultured in contact with WT neurons, related to Figure 1.
Violin plots indicate the median (dashed line) and 25th and 75th percentiles (dotted lines) of Synapsin1/2 puncta density (A) and puncta area (B) of neurons cultured in contact with WT or KO cortical astrocytes. Data are indicated as mean±SEM. ***$p<0.001$ by Mann-Whitney test. Analyses were performed on n>49 neurons per experimental group from N=6 biological replicates. Samples derived from 2 independent experiments.

Figure S3

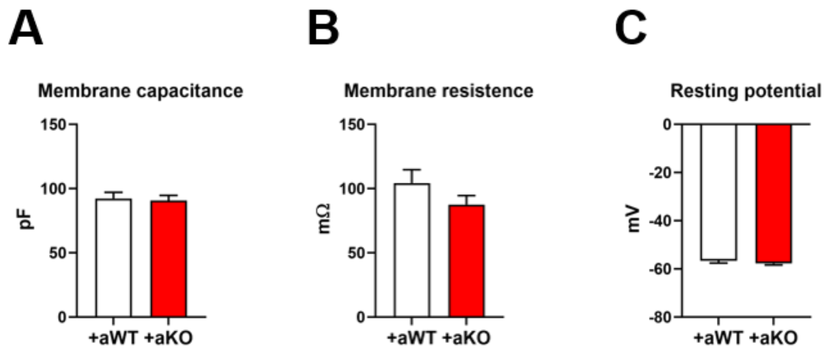

Figure S3. Passive properties of recorded WT neurons cultured with WT or KO astrocytes, related to Figure 1. Membrane capacitance (A), Membrane Resistance (B) and Resting Potential (C) in neurons cultured with WT astrocytes (n=27) and KO astrocytes (n=35).

Figure S4

A

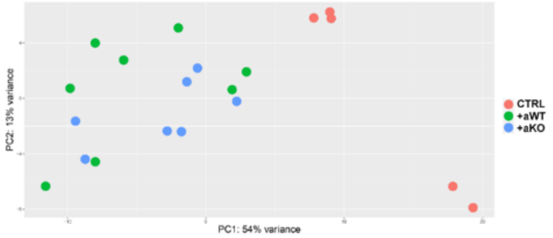

B

	p.adj<0.1	p.adj<0.1 LFC>|1|	p.adj<0.1 LFC>1	p.adj<0.1 LFC<-1	p.adj<0.05	p.adj<0.05 LFC>|1|	p.adj<0.05 LFC>1	p.adj<0.05 LFC<-1
+aWT vs CTRL	3239	545	437	108	2493	545	437	108
+aKO vs CTRL	3808	717	573	144	2890	716	572	144
+aKO vs +aWT	401	25	24	1	216	19	18	1

C

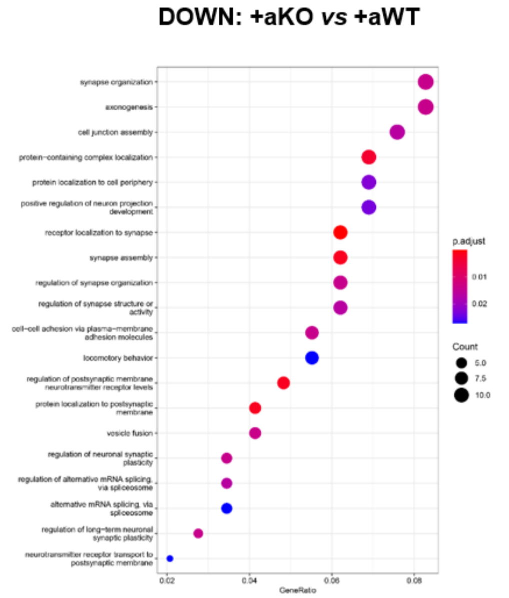

D

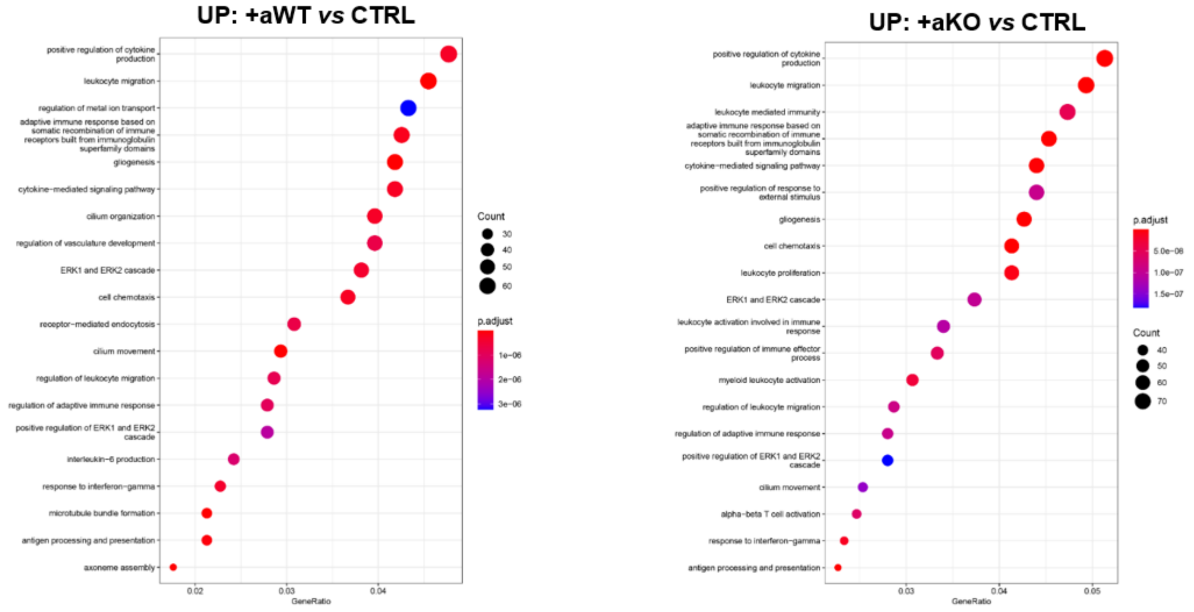

Figure S4. ***Mecp2* KO astrocytes alter gene expression in WT co-cultured neurons, related to Figure 2**.

(A) Principal Component Analysis (PCA) of RNA Seq-data. Gene expression was investigated in neurons cultured alone (CTRL), in neurons cultured with WT (+aWT) or *Mecp2* KO (+aKO) astrocytes. PCA was performed using normalized RNA-Seq data. A clear difference was observed between CTRL and +aWT/+aKO samples. **(B)** The table reports the number of differentially expressed genes (DEGs) in the different comparisons, filtering on the basis of FDR adjusted p-value (p.adj) and log2 fold change (LFC). **(C)** Enrichment analysis data of +aWT *vs* +aKO comparison with upregulated DEGs at p.adj<0.1 (see Table S1), showing the top 20 significant GO terms (biological process). Size and colour of each dot represent -log$_2$ of FDR and number of genes associated with each term, respectively, according to the scale indicated in the figure. **(D)** Enrichment analysis data of +aWT *vs* CTRL (left) and +aKO *vs* CTRL comparisons (right) with upregulated DEGs at p.adj<0.05 (see Table S7 and S8, respectively), showing the top 20 significant GO terms (biological process). Size and colour of each dot represent -log$_2$ of FDR and number of genes associated with each term, respectively, according to the scale indicated in the figure.

Figure S5

A

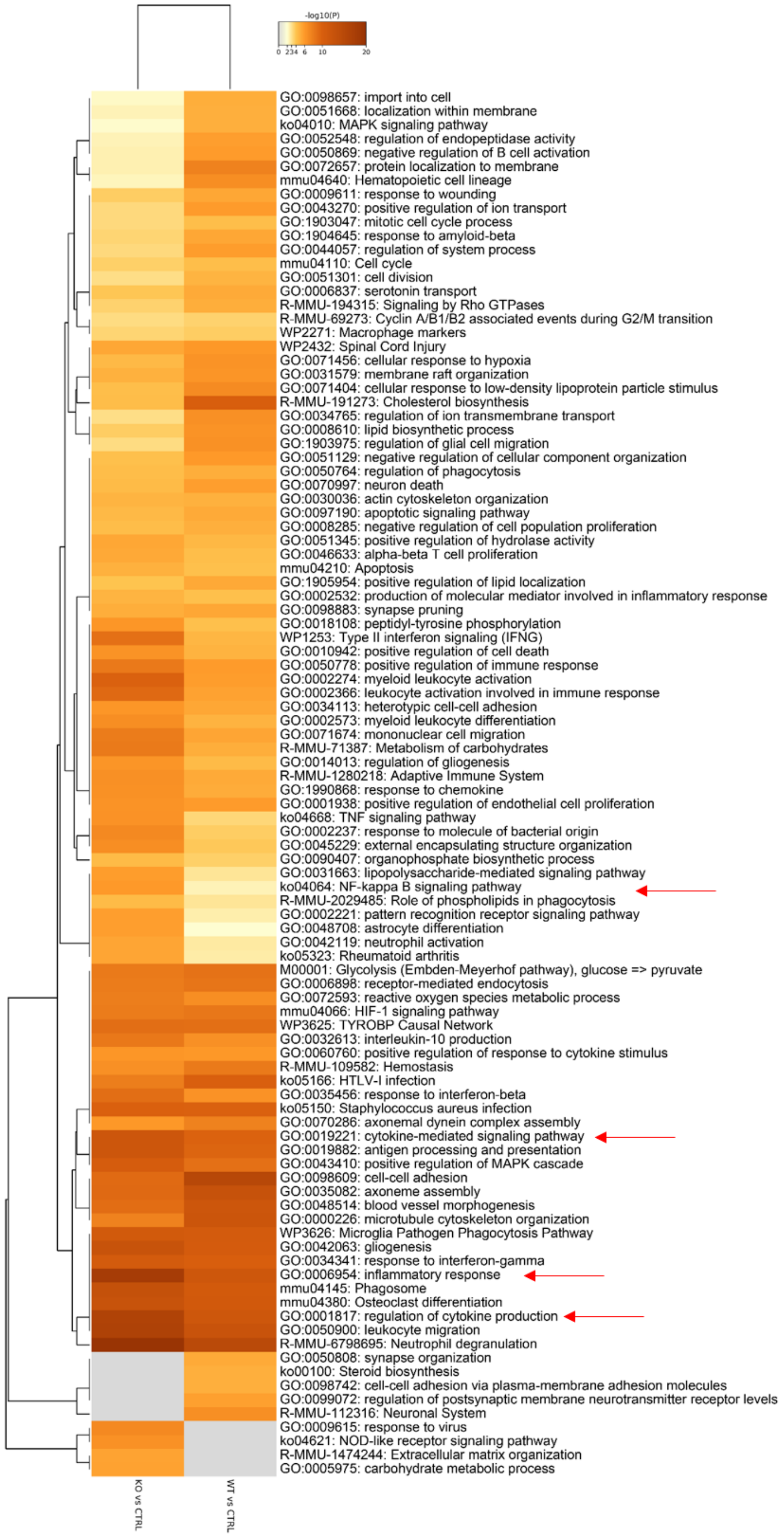

Figure S5. Metascape analysis on upregulated DEGs indicates that pathways related to inflammation are more represented in the +aKO *vs* CTRL with respect to the +aWT *vs* CTRL comparison, related to Figure 3. Heatmap of top 100 enriched terms across the two gene lists, colored by p values.

Figure S6

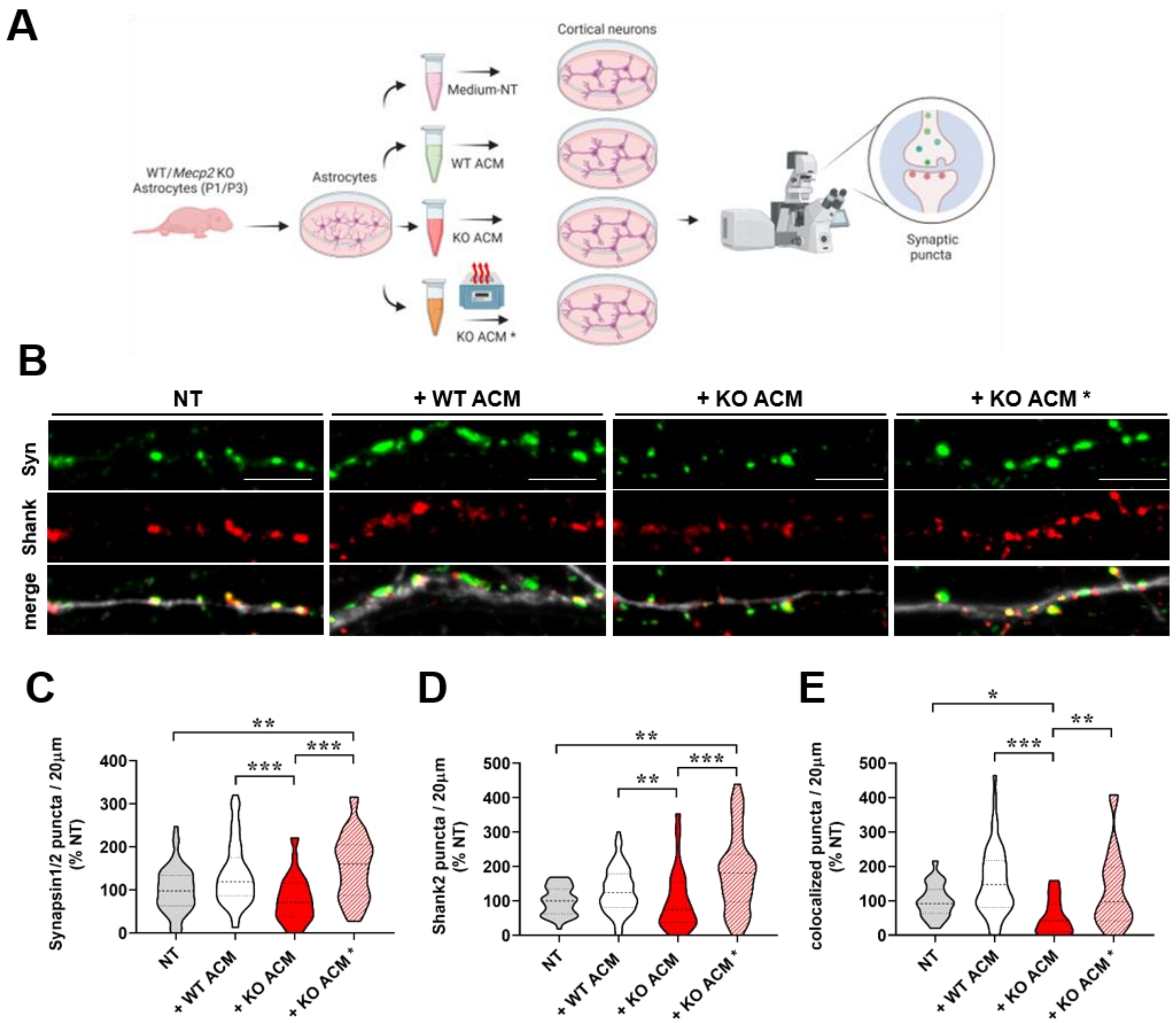

Figure S6. Astrocyte conditioned medium from KO astrocytes (KO ACM) affects synapses, related to Figure 5. (A) Experimental design overview. **(B)** Representative images of primary branches from WT neurons (DIV14) immunostained for Synapsin1/2 (green), Shank2 (red) and their merge with MAP2 (white). KO ACM* indicates heat-inactivated ACM. Scale bar = 5 µm. **(C-E)** Violin plots indicate the median (dashed line) and 25th and 75th percentiles (dotted lines) of Synapsin1/2 (C), Shank2 (D) and colocalized puncta number (E). Values for puncta number are expressed as percentages of neurons treated with empty medium (NT). *p<0.05, **p<0.01, ***p<0.001 by Kruskal-Wallis test followed by Dunn's post hoc test. Analyses were performed on n>24 neurons per experimental group from N>4 biological replicates. All data derived from at least 2 independent experiments.
